# Nonlinear EHD instability of two viscoelastic fluids under the influence of mass and heat transfer

**DOI:** 10.1038/s41598-023-27410-z

**Published:** 2023-01-07

**Authors:** Galal M. Moatimid, Marwa H. Zekry, Doaa A. Ibrahim

**Affiliations:** 1 Department of Mathematics, Faculty of Education, Ain Shams University, Roxy, Cairo, Egypt; 2 Department of Mathematics and Computer Science, Faculty of Sciences, Beni-Suef University, Beni-Suef, Egypt

**Keywords:** Mathematics and computing, Physics

## Abstract

This study attempts to provide an approach to studying the nonlinear stability of a vertical cylindrical interface between two Oldroyd-B prototypes. An unchanged axial electric field influences the system, and porous medium, and the effects of heat and mass transfer (MHT) are considered. Hsieh's modulation and the viscous potential flow (VPT) are used to abbreviate the mathematical analysis. The viscoelastic Oldroyd-B model significant role in geothermal, engineering and industrial enhancement motivated us to carry out this in-depth investigation. The methodology of the nonlinear technique depends mainly on solving the linear equations of motion and applying the appropriate nonlinear boundary conditions. Numerous non-dimensional physical numbers are exposed using a non-dimensional technique. The stability conditions are theoretically achieved and numerically verified. As a limiting case, the linear dispersion equation is accomplished, and a set of stability diagrams is reachable. Together with the nonlinear stability method, a Ginzburg–Landau equation is derived. Subsequently, both theoretical and numerical methods are used to achieve the nonlinear stability criteria. Furthermore, a precise perturbed approach for surface deflection is achieved theoretically and numerically using the Homotopy perturbation method and the extended frequency conception. Along with the linear approach, it is found that the structure becomes unstable by the Laplace, Reynolds, Weber, and elasticity quantities as well as the linear MHT parameter. Furthermore, the stability zones are enhanced in the nonlinear instability approach.

## Introduction

Electrohydrodynamics (EHD) is an area of Fluid Mechanics regarding electrical strength impacts^1^. It could be regarded as an issue that deals with moving fluids in an electric field. Fundamentally, it is a combination of these two areas because many of the most exciting challenges in EHD include both the influence of liquid motion and the impact of electric strength. On the other side, EHD incorporates a complicated interaction of internal, viscous, and electric forces. It produces attractive phenomena in processing equipment in inkjet printing, drops sparing, and other applications. Rayleigh was the first to study charged droplet instability over a century ago; therefore, EHD has been a hot topic. A review article was constructed to give an integrated picture of EHD^2^. The interface stability separating two layers of dielectric liquids in the existence of an applied voltage in addition to imposed fluid fields was examined. Several pioneering works were done, and many researchers have been studying the EHD stability of the interface dividing two superimposed liquids in both bounded and unbounded channels^3^. Using the surface-connected prototype, the EHD instability of two superimposed liquids in a channel was scrutinized^4^. The EHD stability of an incompressible viscous fluid jet was presumed by a uniform longitudinal electric strength, using a weakly nonlinear approach^5^. The nonlinear boundary conditions were employed along with the linear fundamental equations of motion. The stability of an interface separating different liquids with electrical relaxation time was investigated in depth. The nonlinear EHD of capillary surface waves was investigated^6^. The effect of a longitudinal periodic field on a streaming flow passing through three coaxial vertical cylinders was investigated^7^. Coupled Mathieu equations were conducted in their approach. They considered a standard example of surface displacement. The behavior of an electric strength on two cylindrical boundaries was studied^8^. Through complex quantities, the method produced differential equations with damped terms. For more convenience, these equations were combined in light of the symmetrical and anti-symmetric perturbations. Three coupled magnetic fluid interfaces were analyzed for nonlinear azimuthal stability^9^. Their nonlinear method was constructed primarily on resolving the linear regulating equations of motion and applying the associated nonlinear boundary conditions. A streaming dielectric liquid jet EHD stability was conducted. The system was examined to see if a constant axial electrostatic field had entered it^10^. From their process, several non-dimensional numbers were produced. Their research gave researchers a strong foundation for understanding how viscous liquid jets break up and become unstable in the presence of electric strength. Because of how much the electric strength influences things, the current technique is employed in this direction.

Today, there is a growing and substantial quantity of literature on Newtonian fluid cylinder instability. Viscoelastic liquids are non-Newtonian liquids that demonstrate both viscous and elastic properties under certain conditions. Walters, Rivlin-Ericksen, Maxwell, Kelvin, Oldroyd, and others suggested many forms of viscoelastic models. Fluid flows in geophysics, such as polymer distribution, particle filtration from fluids, and lubrication, are frequently represented as viscoelastic flows. The complicated structure of the fundamental performance of such fluids makes further complicated things for viscoelastic jets^11^. The non-Newtonian liquid takes part in a wide range of productions and scientific disciplines. The Oldroyd-B is considered a good model to investigate further significant experiments among several prototypes that have been employed to illustrate the performance of non-Newtonian liquids. This model was the most successful for comparing the reactions of numerous viscoelastic liquids, since it can predict stress relaxation, creep, and normal stress changes. It covers the Maxwell and viscous liquid simulations as specific cases. Industries are interested in the motion of fluids in the surroundings of a moving body. One of the really significant and fascinating movements was the flowing through revolving cylinders which have different uses in the food sector. Non-Newtonian liquids possess mechanics that are both practically and theoretically significant. The subject was theoretically interesting since the linear instability evaluation of these liquids was helpful in highlighting the properties under certain conditions^12^. When HMT plays a significant role in establishing stream stability, injector-sparing qualities were examined. The Kelvin–Helmholtz instability (KHI) of an annular Oldroyd-B was examined^13^. The curve of the maximum development rate versus the wave numeral was obtained and analyzed in numerous situations. Because of its important role in numerous practical applications of the use of Oldroyd-B viscoelastic fluid, the current work was conducted in relation to this topic. A commonly employed technique for insulating wires for mechanical strength and environmental regulation was wire coating. In an experiment, feed-forward artificial neural networks were utilized to soak the cables in an Oldroyd 8 constant fluid while maintaining a constant pressure gradient in order to analyze the nonlinear wire covering^14^.

Many different industrial projects and natural systems used porous media. Predicting the features of the fluid flow and computing the pressure drop over a permeable material was often essential for the construction of engineering systems. Instability in a permeable medium was vital for energy transfer from ordinary gas to hydrogen, and most importantly, in assessing the ability to exist in subsurface gas storage. In view of mixed gases inside storage, the interface stability led to correcting the speed rate of injection and withdrawal of environmental gases. The interface stability of compressible liquids in permeable media was investigated^15^. The problem was handled more effectively when limited flows were considered. Additionally, their approach was especially important when dealing with two-dimensional gas fluxes in storage facilities. It has a wide range of practical applications in numerous areas, such as geophysical science^16^, incineration machinery, oil manufacturing, and petrochemical industries regarding the recovery of oil on or after the concentrates of the stored rocks. The KHI in porous media has been of considerable interest to researchers, particularly in petroleum reservoirs. The necessary requirements for the shifting interface instability of two incompressible liquids flow in permeable media were described^17^. In this case, the two liquids are of Newtonian behavior, and Darcy’s law could be applicable. As the liquid gradually increased across the pore spaces of the rock, the gross influence was addressed by Darcy’s law. It indicated that the normal viscosity parameter in the fundamental equation may be represented by the factor $\mu \,\underline{u} /\kappa$, where $\mu$ is the liquid viscosity, $\kappa$ is the permeability of the medium, and $\underline{u}$ is the velocity of the liquid. The KHI of incompressible fluids passing across permeable media was widely examined^18^. When considering collision effects, the media, natural particles, and the stability of a planar interface between two superposed composite plasmas with uniform densities in permeable media were scrutinized. Sharma^19^ evaluated how a horizontally magnetic strength affects the KHI of two superimposed plasmas and discovered that the growth rate was lowered by medium porosity. The KHI at the interface concerning a permeable material filled with a poorly conducting fluid was investigated^20^. The impacts of the magnetic and electric strengths, permeability, and slipping factor in lowering the growth rate were presented. These appeared to have a potential to change the underlying instabilities in several current applications. Compressibility impacts on the Rayleigh–Taylor instability (RTI) and KHI connecting two immiscible liquids moving over a permeable material were methodologically described^21^. Remember that the current study investigates the effects of a uniform tangential electrostatic field as well as the interfacial nonlinear stability in the presence of permeability. The relevance of the porous interaction has led to the advantage of this approach in the current study.

In the VPF, when the vorticity vanishes, the viscous part in the Navier–Stokes equations will disappear, but the viscous stresses do not^22^. On the other side, the tangential stresses are ignored, and viscosity was incorporated only throughout the normal stress balancing. The no-slip criterion at the border was not satisfied with this theory, allowing two-dimensional solutions to match three-dimension responses. The VPF of capillary instability was investigated^23^. The VPF was a better-quality approach to estimating the analytic solution than the ideal prototype. An examination of capillary stability was carried out on viscoelastic liquids of Maxwell classes. It was found that the growth rates for viscoelastic liquids were faster than the Newtonian ones^24^. The instability of a fluid jet in incompressible gases and fluids was analyzed. They looked at both KHI and capillary instability and found that KHI cannot happen in a vacuum, in contrast with capillary instability. Along with the VPF, the RTI of a cylindrical flow was examined^25^. The growth rate of the considered problem produced a quadratic formula that served as the dispersion relationship. The VPF was used to examine the stability of a horizontal magnetic sheet. Coupled Mathieu differential equations, with complex coefficients and damped terms were originated^26^. The nonlinear azimuthal instability requirements of connected interfaces involving three magnetic liquids were studied^9^. They took a nonlinear methodology, relying on the solution of the underlying linear equations of motion and the applicable nonlinear border restrictions. The nonlinear instability of a cylindrical-shaped interface connecting two incompressible viscoelastic liquids was scrutinized^27^. A time-periodic field acted on the system as well as the simplified formulation of the VPF was adopted to reduce the mathematical manipulation. The current research paper was based on this idea in compliance with the simplicity of making a VPT allowance. The existing work was undertaken in light of this idea in compliance with the simplicity of making a VPT permission. As well known, the mass and the quantity of heat transmission have a direct relationship. It is impossible to imagine this magnificent nature, with people, animals, jungles, the sea, and rivers, without heat transfer. Even though heat transfer is involved in the principle of heat transmission from one location to another. Heat transfer was responsible for the transfer of solar energy from the sun to the earth. Consequently, the current work is done under the effect of MHT. On this phenomenon, recent research was discovered^28–33^.

Given the significance of the aforementioned highlights, the purpose of this article is to investigate the mechanisms of a non-Newtonian liquid cylinder from a nonlinear temporal stability approach of the liquid viscoelastic jet. The Oldroyd model^34^ represents a viscoelastic jet constitutive behavior. The current study may provide a useful starting point for additional inquiry into the instability and breakup of viscoelastic fluid jets. A full approach to the theoretical prototype, together with the fundamental equations, reveals a nonlinear characteristic dispersion relationship between the growth rate and wave numeral of viscoelastic fluid jet. In conclusion, since liquid jet-breaking offers a much more mechanism of descriptive physical significance than experimental formulae, the analytical method of liquid jets was more universally applicable. In air-blast atomizers, the breakdown of liquid jets was a complicated process. The liquid jet was susceptible to a variety of instabilities, such as capillary, helical, KHI, etc., depending on the atomizer operating flow parameters, and numerous processes of the liquid core and attachments breaking apart have been found in prior research. These processes were related to the development of disturbances waves on the liquid–air interface, which occurs downstream of the injector and, once a critical waveform was achieved, cause the liquid jet to fragment into ligaments and/or droplets^35^ and^36^.

The major objective of the current study is to help readers to look for appropriate responses to the following questions:

1. What are the criteria of the linear stability approach?
2. How many physical non-dimensional numbers are present throughout the linear technique?
3. What are the effects of the nondimensional physical numbers on the stability configuration?
4. What is the approach of the nonlinear stability sense?
5. What is the formula for the approximate solution to the interface displacement?

For ease of understanding, the outstanding physical configuration will be established as follows: Section “Methodology of the model” introduces the approach of the physical prototype. The fundamental controlling equations of motion and the relevant nonlinear border restrictions are involved in this Section. In section “Linear stability methodology”, the relevant linear stability methodology is introduced. Additionally, a set of graphs is drawn in order to depict the influences of the physical non-dimensional factors in the instability information. The nonlinear aspects in addition to the relevant numerical estimation are introduced in section “Nonlinear stability methodology”. An approximate form of the interface displacement via the HPM and the expanded nonlinear frequency approach is provided in section “Estimated surface departure formulation”. Furthermore, two dimensions of the interface displacement are displayed. Finally, in section “Concluding remarks”, concluding remarks are formed based on the main ideas.

## Methodology of the model

**Figure 1:**
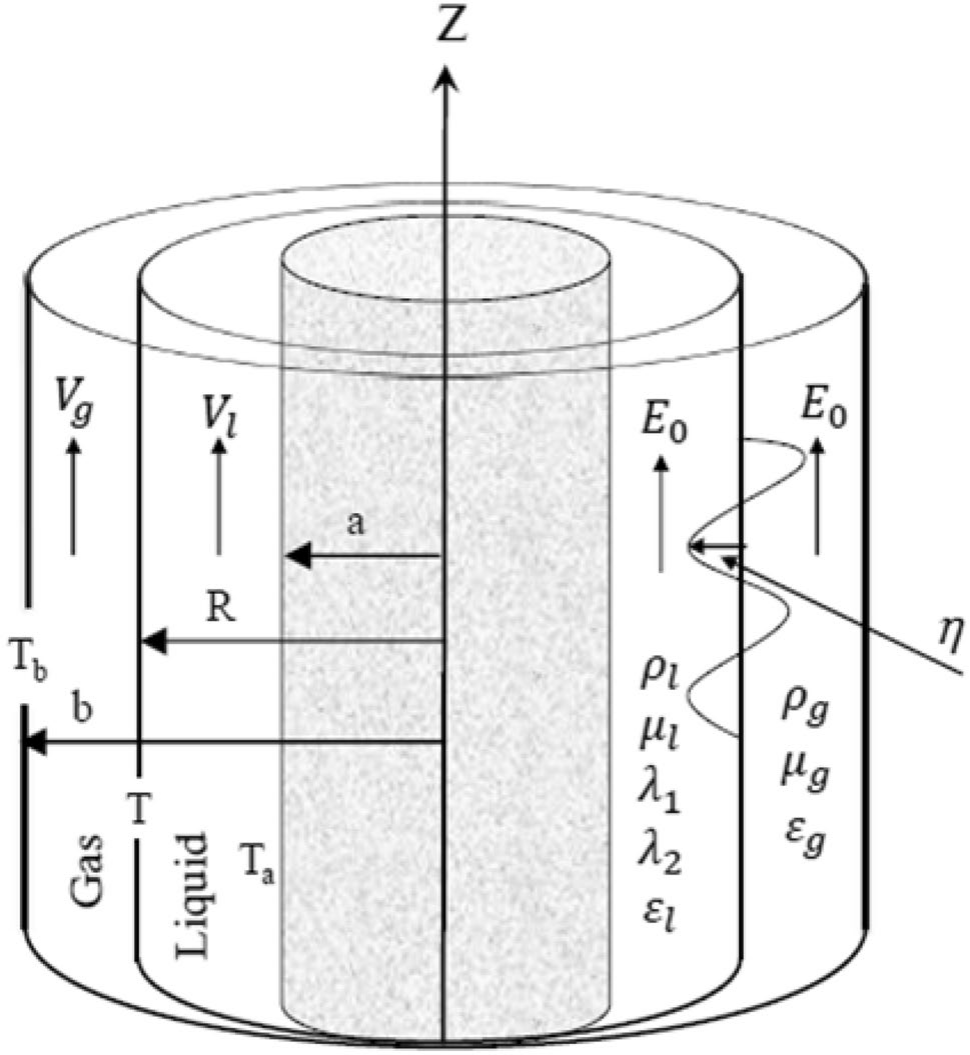
Illustration of the theoretical prototype.

Two homogeneous, incompressible, and dielectric-flowing liquids make up the structure under consideration. The theoretical system consists of three cylindrical surfaces. The inner cylinder is a solid one, and it is raised to a uniform temperature. The flow is governed by Darcy's law throughout porous mediums. For ease of reference, the permeability of the two fluids is simply referred to as. Scientific evidence supports Darcy's law, an experimental hypothesis that describes the creeping flow of Newtonian liquids in porous media. A non-Newtonian viscoelastic fluid obeying an Oldroyd-B flow occupies the inner cylindrical annular liquid. This liquid is characterized by certain constants, which are addressed as retardation and relaxation times in addition to the viscosity parameter. Viscoelasticity is vital for understanding how blood flow behaves, and the constitutive model of Oldroyd-B fluid can be applied in this area. It contains relaxation and retardation times, as two important characteristics of viscoelastic fluid. Restoring an unstable system to equilibrium is typically referred to as relaxing. A relaxation time can be used to categorize each relaxation step. Electrical conductivity and the dielectric relaxation time are strongly connected. It is an indicator of how long it takes for an electrical charge to be neutralized in a semiconductor. Metals have a short relaxation time, whereas semiconductors and insulators can have a long one. Retardation, also known as "delay of the elasticity," is the delayed response to an applied force or stress. Ideal elastic materials exhibit an instantaneous deformation with the application of a tension resembling a leap and an instantaneous reformation upon the removal of the load. For instances of viscoelastic materials, this elastic behavior happens after a specific amount of time. Numerous polymer industries, blood flow, etc. use relaxation/retardation time as a crucial characteristic of viscoelastic fluid flows. A portion of the energy in the cardiovascular system is stored because the blood is elastic, a portion is lost as heat because the blood is viscous, and a portion is involved in blood mobility^37^. A viscous gas occupies the outer cylinder and possesses another viscosity constant. In the undisturbed situation, these two phases are divided by a hypothetical cylindrical interface. This interface is assumed to have another uniform temperature. Finally, the inner cylinder is a solid one and it is raised to distinct constant temperatures. The two phases basically have uniformly various velocities. A longitudinal unchanged electric strength is portrayed in the system. Therefore, the cylindrical coordinates are more convenient for this clarification. The surface tension parameter is taken into the explanation. To facilitate the mathematical manipulation, the MHT is analyzed in accordance with Hsieh’s explanation^38^ and^39^. This oversimplification results in a single parameter in the linear stability approach, as will be seen later. The nonlinear methodology, on the other side, yields only two parameters to control MHT. Accordingly, this approach would substantially facilitate the relationship experiment. Alongside the negative $z -$ path, the gravitational force is considered. The fluid jet is stable for all asymmetric types, but unstable for the axisymmetric way, as demonstrated by several researchers such as Chandrasekhar^40^. Consequently, the axisymmetric mode is the most interesting one in this study. Therefore, the radiating symmetry is merely reflected. Henceforth, all fundamental amounts will be unrestricted with the angular position. A similar problem with the Walters-B fluids has been recently analyzed^41^. For more explanation, Fig. 1 describes the physical and theoretical model.

The distributed cylindrical surface formulation can be expressed as:

1 $$r = R + \eta (z,t),$$

where $\eta (z;t)$ is the surface movement.

Consequently, the interface may be given following a small deviation from the equilibrium situation. It could be characterized as:

2 $$S(r,z;t) = r - R - \eta (z,t).$$

It is possible to formulate the unit normal vector as:

3 $$\underline{n} = \nabla S/\left| {\nabla S} \right| = (\underline{e}_{r} - \,\eta_{z} \,\underline{e}_{z} )(1 + \eta_{z}^{2} )^{ - 1/2} .$$

The Oldroyd–B model of viscoelastic fluids is used in this structure, where the constants determine the characteristics of these fluids. The Cauchy stress tensor $\underline{\underline{\tau }}^{vis}$ of this model can be signified by Oldroyd^42^ and^43^:

4 $$\underline{\underline{\tau }}^{vis} = - P\underline{\underline{I}} + \underline{\underline{\tau }} ,$$

where $P$ is the hydrostatic pressure, $\underline{\underline{I}}$ denotes the identified tensor, and $\underline{\underline{\tau }}$ symbolizes an additional tensor that can be described as:

5 $$\underline{\underline{\tau }} + \lambda_{1} \left( {\frac{{d\underline{\underline{\tau }} }}{dt} - \underline{\underline{\tau }} .\,\underline{\underline{N}} - \underline{\underline{N}}^{T} .\,\underline{\underline{\tau }} } \right) = \mu_{l} \left( {\underline{\underline{A}} + \lambda_{2} \frac{{d\underline{\underline{A}} }}{dt} - \underline{\underline{A}} \,.\,\underline{\underline{N}} - \underline{\underline{N}}^{T} .\,\underline{\underline{A}} } \right).$$

The strain tensor rate is also identified as:

6 $$\underline{\underline{A}} = \underline{\underline{N}} + \underline{\underline{N}}^{T} ,$$

where $$\underline{\underline{N}} = \nabla \underline{v}$$.

The Oldroyd-B model is provided by incorporating the traditional Newtonian prototype as a particular situation $\lambda_{1} = \lambda_{2} = 0$. Furthermore, it is shortened to the Maxwell model at $\lambda_{2} = 0$. Darcy’s law states that the decreased pressure is caused by a frictional drag in a viscous Newtonian liquid flowing at a lower speed and directly proportional to velocity. The relationship between pressure drops and the speed of a viscoelastic liquid in a permeable medium may be characterized as previously given^44^ in contrast to the Oldroyd-B constitutive relationships.

7 $$\left( {1 + \lambda_{1} \frac{\partial }{\partial t}} \right)\nabla p = - \frac{{\mu_{l} }}{\xi }\left( {1 + \lambda_{2} \frac{\partial }{\partial t}} \right)\underline{v}_{{}} ,$$

The localized conservation of momentum balancing is provided by the evidence of the balancing of forces working on a volume amount of liquid.

8 $$\rho \left( {\frac{{\partial \underline{v} }}{\partial t} + (\underline{v} .\nabla )\underline{v} } \right) = - \nabla P + \rho g\underline{e}_{z} + \nabla .\underline{\underline{\,\tau }} + \underline{\Upsilon } \,$$

Because the pressure gradient in Eq. (7) indicates that the flow resistance in the volume of the permeable substance can be measured, the movement resistance provided by the solid matrix is indicated by $\underline{\Upsilon }$. The vector $\underline{\Upsilon }$ can be determined from Eq. (7) to fulfill the following equation as^45^:

9 $$\left( {1 + \lambda_{1} \frac{\partial }{\partial t}} \right)\underline{\Upsilon } = - \frac{{\mu_{l} }}{\xi }\left( {1 + \lambda_{2} \frac{\partial }{\partial t}} \right)\underline{v} .$$

Combining Eq. (8) with Eq. (9), as shown in^46^, one gets

10 $$\left( {1 + \lambda_{1} \frac{\partial }{\partial t}} \right)\left[ {\rho \left( {\frac{{\partial \underline{v} }}{\partial t} + (\underline{v} .\nabla )\underline{v} } \right) + \nabla P - \rho g\underline{e}_{z} - \nabla .\,\underline{\underline{\tau }} \,} \right]\, = - \frac{{\mu_{l} }}{\xi }\left( {1 + \lambda_{2} \frac{\partial }{\partial t}} \right)\underline{v} .$$

Because the convective part $(\underline{v} .\nabla )\underline{v}$ and the viscous part $\nabla .\underline{\underline{\,\tau }}$ may be ignored in the Darcy model, the momentum equation of the Oldroyd-B version with a permeable form can be calculated as

11 $$\left( {1 + \lambda_{1} \frac{\partial }{\partial t}} \right)\left( {\rho \frac{{\partial \underline{v} }}{\partial t} + \nabla P - \rho g\underline{e}_{z} \,} \right)\, = - \nu_{l} \left( {1 + \lambda_{2} \frac{\partial }{\partial t}} \right)\underline{v} .$$

Equation (11) can now be rewritten as

12 $$\rho \frac{{\partial \underline{v} }}{\partial t} + \nabla P - \rho g\underline{e}_{z} \, = - \nu_{l} \left( {1 + \lambda_{1} \frac{\partial }{\partial t}} \right)^{ - 1} \left( {1 + \lambda_{2} \frac{\partial }{\partial t}} \right)\underline{v} .$$

The Darcy coefficient is equal to $\nu_{l} = \mu_{l} /\xi$.

The general stress tensor is constructed of two elements. The earliest element is the viscoelastic tensor of the Oldroyd-B prototype which is produced in Eqs. (4) and (5). The mixture of Eqs. (4) and (5) generates:

13 $$\underline{\underline{\tau }} = \mu_{1} \left( {1 + \lambda_{1} \frac{\partial }{\partial t}} \right)^{ - 1} \left( {1 + \lambda_{2} \frac{\partial }{\partial t}} \right)\,\underline{\underline{A}} .$$

The continuity equation becomes

14 $$\nabla .\underline{v}_{j} = 0\,,\,\,\,\,\,\,\,\,\,\,\,\,\,\,\,\,j = l,g,$$

Based on the basic assumptions of the VPF, one might presume that the liquids are irrotational. Accordingly, the velocity potentials exist as the distribution $\phi_{j} (r,z;t)$ such that

15 $$\underline{v}_{j} = V_{j} \underline{e}_{z} + \nabla \phi_{j} = \frac{{\partial \phi_{j} }}{\partial r}\underline{e}_{r} + \left( {V_{j} + \frac{{\partial \phi_{j} }}{\partial z}} \right)\underline{e}_{z} ,\,\,\,\,\,\,\,\,\,j = l,g.$$

Owing to the incompressibility circumstance, the potential $\phi_{j}$ should verify the following Laplace equation:

16 $$\nabla^{2} \phi_{j} = 0\,.$$

Many investigators have proven that the solutions of distribution functions are typically based on an understanding of natural conditions^9^. As a result of the standard mode approach, one may presume

17 $$\phi_{j} (r,z;t) = \hat{\phi }_{j} (r;t)e^{ikz} + c.c.\,$$

The solution of Eq. (16) then becomes

18 $$\hat{\phi }_{j} (r;t) = A_{j} (t)I_{0} (kr) + B_{j} (t)K_{0} (kr),$$

where $A_{j} (t)$ and $B_{j} (t)$ are unspecified time-dependent formulas. They can be calculated employing nonlinear border circumstances. The modified Bessel functions of zero order of the first and second kind, correspondingly, are signified by $I_{0} (kr)$ and $K_{0} (kr)$.

The pressure is calculated by explaining Eq. (8). Following the approach of Bernoulli's equation, one can find the principal momentum equation by direct integration.

19 $$P_{j} = - \rho_{j} \left( {\frac{{\partial \phi_{j} }}{\partial t} + i\,k\,V_{j} \phi_{j} } \right) - \upsilon_{j} \phi_{j} - (\rho_{j} g + \upsilon_{j} V_{j} )z + g_{j} (t).$$

The established Maxwell’s formulae must be characterized owing to the overall electrostatic impact on the problem at hand; for instance, see Melcher^47^. Melcher purported a comprehensive book that contained a completed inspection of the surface-coupled EHD and Magnetohydrodynamics (MHD) structures. In the contemporary case, just the impression of a tangential electrostatic strength is reflected. Consequently, in the present study, the presence of magnetic strength can be ignored. Depending on all these assumptions, along with the quasi-static approximation, Maxwell’s formulae are reduced to:

20 $$\nabla .\varepsilon_{j} \underline{E}_{j} = 0\,,$$

and

21 $$\nabla \times \underline{E}_{j} = \underline{0} .$$

Subsequently, the electric strength $\underline{E}_{j}$ could be denoted with a scalar function $\psi_{j} (r,\,z;\,t)$, as

22 $$\underline{E}_{j} = E_{0\,} \underline{e}_{z} + \nabla \psi_{j} .$$

The outcome is Eqs. (20) and (21), the potential of the electric field follows Laplace’s equation:

23 $$\nabla^{2} \psi_{j} = 0.$$

As stated in Eq. (17), the electric potentials could be expressed as:

24 $$\hat{\psi }_{j} (r;t) = C_{j} (t)I_{0} (kr) + D_{j} (t)K_{0} (kr),$$

where $C_{j} (t)$ and $D_{j} (t)$ are unspecified time-dependent effects that will be assessed later.

### Nonlinear border criteria

*At the solid cylinders (*$r = a$* and *$r = b$*).*

- The fluid normal velocities should always be eliminated; hence, the following prerequisites should be met.:
  25 $$\frac{{\partial \phi_{l} }}{\partial r} = 0\,\,\,\,\,\,\,\,\,\,\,\,\,\,\,\,\,\,\,\,\,\,\,\,\,\,\,\,r = a,$$

and

26 $$\frac{{\partial \phi_{g} }}{\partial r} = 0\,\,\,\,\,\,\,\,\,\,\,\,\,\,\,\,\,\,\,\,\,\,\,\,\,\,\,\,r = b.$$

- The tangential elements of the electric potential have to be removed, which means
  27 $$\frac{{\partial \psi_{l} }}{\partial r} = 0\,\,\,\,\,\,\,\,\,\,\,\,\,\,\,\,\,\,\,\,\,\,\,\,\,\,\,\,r = a,$$

and

28 $$\frac{{\partial \psi_{g} }}{\partial r} = 0\,\,\,\,\,\,\,\,\,\,\,\,\,\,\,\,\,\,\,\,\,\,\,\,\,\,\,\,r = b.$$

*At the free cylindrical boundary *$r = R + \eta (z,t)$.

- The preservation of mass and energy through the surface of separation was achieved by using the abbreviated modulation, which was first suggested by Hsieh^38^ and^39^. This created the following requirements.:
  29 $$\left\| {\rho_{j} \left( {\frac{\partial S}{{\partial t}} + \underline{{v_{j} }} .\nabla .S} \right)} \right\| = 0,$$

where $\left\| * \right\| = *_{2} - *_{1}$ indicates, correspondingly, the difference between the interior and exterior liquid phases.

- According to Hsieh^38^ and^39^., it is expected that the immediate location of the interface has a significant impact on how much latent heat is released. Consequently, the interface requirement for heat exchange is expressed as:
  30 $$L\,\rho_{l} \left( {\frac{\partial S}{{\partial t}} + \underline{v}_{l} \,.\nabla S} \right) = f(\eta )$$

where $f(\eta )$ signifies the net heat flow from the interface and may be formulated as:

31 $$f(\eta ) = \frac{{K_{g} (T - T_{b} )}}{(R + \eta )(\ln \,b - \ln (R + \eta ))} - \frac{{K_{l} (T_{a} - T)}}{(R + \eta )(\ln \,(R + \eta ) - \ln \,a)}.$$

When expanding $f(\eta )$ in the around $\eta = 0$, one gets

32 $$f(\eta ) = f(0) + \eta \,f^{\prime}(0) + \frac{1}{2}\eta^{2} f^{\prime\prime}(0) + \frac{1}{6}\eta^{3} f^{\prime\prime\prime}(0) + ...$$

The quantity $f(0)$ denotes the net heat flow from the interface into the liquid zones. Since $f(0)$ is assumed to be zero, the following relationship can be derived:

33 $$\frac{{K_{g} (T - T_{b} )}}{R(\ln \,b/R)} = \frac{{K_{l} (T_{a} - T)}}{R(\ln \,R/a)} = G,$$

where $G$ is constant, signifying that the heat flux throughout the interface connects the two liquids. It was considered to be identical in the equilibrium condition.

Equations (1), (31), and (32) are now substituted into Eq. (30), yielding the following equation:

34 $$\rho_{l} \left[ { - \frac{\partial \eta }{{\partial t}} + \left( {\frac{{\partial \phi_{l} }}{\partial r} + i\,k\,V_{l} \eta - k^{2} \eta \phi_{l} } \right)} \right] = \alpha_{1} \eta + \alpha_{2} \eta^{2} + \alpha_{3} \eta^{3} .$$

where the constants $\alpha_{1} ,\,\alpha_{2} \,,\,{\text{and}}\,\,\alpha_{{3}}$ may be listed as follows:

$$\alpha_{1} = \frac{G}{R\,L}\left( {\frac{1}{{\ln \left( {{b \mathord{\left/ {\vphantom {b R}} \right. \kern-0pt} R}} \right)}} - \frac{1}{{\ln \left( {{a \mathord{\left/ {\vphantom {a R}} \right. \kern-0pt} R}} \right)}}} \right),\,\,\,\,\alpha_{2} = \frac{1}{2\,R}\left( {2\,\left( {\frac{1}{{\ln \left( {{b \mathord{\left/ {\vphantom {b R}} \right. \kern-0pt} R}} \right)}} - \frac{1}{{\ln \left( {{R \mathord{\left/ {\vphantom {R a}} \right. \kern-0pt} a}} \right)}}} \right) - 3} \right)$$

and

$$-$$

- At the border dividing the two cylindrical liquids, the change in the tangential elements of the electric strength is continuous. Therefore, one finds
  35 $$\underline{n} \times \,\left\| {\underline{E}_{j} } \right\| = \underline{0} .\,$$
- At the connection, the continuously normal electric fluctuation provides
  36 $$\underline{n} \,.\,\left\| {\varepsilon_{j} \,\underline{E}_{j} } \right\| = 0.$$

Replacing Eqs. (18) and (24) into Eqs. (25)-(29) and (35)-(36), it follows that the solutions $\,\phi$ and $\,\psi$ might be exemplified in the form

37 $$\phi_{l} = \frac{{i\left( {I_{1} (ak)K_{0} (kr) + I_{0} (kr)K_{1} (ak)} \right)\left( {m_{1} (\eta (\alpha_{1} + \eta (\alpha_{2} + \alpha_{3} \eta } \right) + \rho_{l} \eta_{t} ) + V_{1} \rho_{l} \eta_{z} )}}{{k\rho_{l} \,\left( {i(I_{1} (kR)K_{1} (ak) - \,I_{1} (ak)K_{1} (kR))) + \eta_{z} (I_{1} (ak)K_{0} (kR) + \,I_{0} (kR)K_{1} (ak))} \right)}}\,,$$

38 $$\phi_{g} = \frac{{i\left( {I_{1} (bk)K_{0} (kr) + I_{0} (kr)K_{1} (bk)} \right)\left( {m_{2} (\eta (\alpha_{1} + \eta (\alpha_{2} + \alpha_{3} \eta } \right) + \rho_{g} \eta_{t} ) + V_{2} \rho_{g} \eta_{z} )}}{{k\rho_{g} \,\left( {i(I_{1} (kR)K_{1} (bk) - \,I_{1} (bk)K_{1} (kR))) + \eta_{z} (I_{1} (bk)K_{0} (kR) + \,I_{0} (kR)K_{1} (bk))} \right)}}\,,$$

39 $$\psi_{l} = \frac{{i\,E_{0} \,(\varepsilon_{l} - \varepsilon_{g} )\,\eta {}_{z}\,}}{\,\Lambda }\left( {\,i\left( {I_{1} (ak)K_{0} (kr) + I_{0} (kr)K_{1} (ak)} \right)\left( {i\left( {I_{1} (bk)K_{0} (kR) + I_{0} (kR)K_{1} (bk)} \right) + \eta_{z} (I_{1} (kR)K_{1} (bk) - I_{1} (bk)K_{1} (kR)} \right)} \right)\,\,,$$

and

40 $$\psi_{g} = \frac{{i\,E_{0} \,(\varepsilon_{l} - \varepsilon_{g} )\,\eta {}_{z}\,}}{\,\Lambda }\left( {\,\left( {I_{1} (bk)K_{0} (kr) + I_{0} (kr)K_{1} (bk)} \right)\left( {\left( {I_{1} (ak)K_{0} (kR) + I_{0} (kR)K_{1} (ak)} \right) - i\eta_{z} (I_{1} (kR)K_{1} (ak) - I_{1} (ak)K_{1} (kR)} \right)} \right).$$

where

$$\begin{gathered} \Lambda = k(\varepsilon_{g} (I_{1} (kR)K_{1} (bk) - I_{1} (bk)K_{1} (kR) - i\eta_{z} (I_{1} (bk)K_{0} (kR) + I_{0} (kR)K_{1} (bk))(I_{1} (ak)K_{0} (kR) + I_{0} (kR)K_{1} (ak) - i\eta_{z} \hfill \\ (I_{1} (kR)K_{1} (ak) - I_{1} (ak)K_{1} (kR)) + \varepsilon_{l} \left( {i(I_{1} (kR)K_{1} (ak) - I_{1} (ak)K_{1} (kR)} \right)\, + \eta_{z} (I_{1} (ak)K_{0} (kR) + I_{0} (kR)K_{1} (ak)) \hfill \\ \left( {i(I_{1} (bk)K_{0} (kR) + I_{0} (kR)K_{1} (bk)} \right)\, + \eta_{z} (I_{1} (kR)K_{1} (bk) - I_{1} (bk)K_{1} (kr)), \hfill \\ \end{gathered}$$

herein $I_{1} (kR)$ and $K_{1} (kR)$ are the modified Bessel differential equations of first and second kind, respectively.

The total stress tensor^48^ is provided as follows:

41 $$\sigma_{ij} = \sigma_{ij}^{vis} + \sigma_{ij}^{elc} ,$$

for the liquid phase, one gets

42 $$\sigma_{ij}^{vis} = - P_{l} \underline{\underline{I}} + \underline{\underline{\tau }} ,$$

where $$\tau_{rr} = 2\mu_{l} M\phi_{lrr} ,\,\tau_{rz} = 2\mu_{l} M\phi_{lrz} ,\,\,\tau_{zz} = 2\mu_{l} M\phi_{lzz} ,$$ and $$M = \mu_{l} \left( {1 + \lambda_{1} \frac{\partial }{\partial t}} \right)^{ - 1} \left( {1 + \lambda_{2} \frac{\partial }{\partial t}} \right)$$,

for the gas phase, one finds

43 $$\sigma^{vis}_{ij} = - P_{g} \underline{\underline{I}} .$$

$\sigma_{ij}^{elc}$ for the two phases yields

44 $$\,\,\,\,\sigma_{ij}^{elc} = \varepsilon \,E_{i} E_{j} - \frac{1}{2}E^{2} \delta_{ij} .$$

- The interface tension-induced discontinuities in the normal stress tensor are the source of the residual border criterion. It could be represented as follows.:
  45 $$\left\| {\underline{n} \,.\,\,\underline{F} } \right\| = T\,\nabla .\,\underline{n} ,\,$$

where $\underline{F}$ is the complete force on the interface that is described as:

46 $$\underline{F} = \left( {\begin{array}{*{20}c} {\sigma_{rr} } & {\sigma_{rz} } \\ {\sigma_{zr} } & {\sigma_{zz} } \\ \end{array} } \right)\left( {\begin{array}{*{20}c} {n_{r} } \\ {n_{z} } \\ \end{array} } \right),$$

where $n_{r}$ and $n_{z}$ are the elements of the unit normal vector $\underline{n}$ and $T$ is the quantity of the interfacial tension.

Therefore, the nonlinear characteristic equation may be expressed as follows:

47 $$\eta_{tt} + (a_{1} + ib_{1} )\eta_{t} + (a_{2} + ib_{2} )\eta_{z} + (a_{3} + ib_{3} )\eta + a_{4} \eta_{zt} + a_{5} \eta_{zz} = G(\eta ),$$

where $G(\eta )$ represents all the nonlinear terms in the displacement function $\eta$. All the constants are supplied in the Appendix to follow the content straightforwardly. It should be emphasized that left-hand side of Eq. (47) demonstrates the linear dispersion relation, allowing us to explain the motion of the cylindrical interface of the linear case. The discussion of this approach is hence the focus of the next Section.

### Abbreviation

In the subsequent table, the characters $L$ and signify the internal and external cylindrical fluids, respectively. where $m$,$l$,$t$ and $K$ denote mass, length, time, and Kelvin, respectively

## Linear stability methodology

It is a respectable impression to look at the stability profile from a linear perspective before moving to the general case. When the nonlinear elements are eliminated from Eq. (47), the dispersion equation is provided for the linear case. The linear dispersion connection can therefore be expressed as:

48 $$\eta_{tt} + (a_{1} + ib_{1} )\eta_{t} + (a_{2} + ib_{2} )\eta_{z} + (a_{3} + ib_{3} )\eta + a_{4} \eta_{zt} + a_{5} \eta_{zz} = 0$$

A consistent wave train solution to Eq. (48) can be presumed with the standard mode approach. Therefore, we may write

49 $$\eta (z,t) = \gamma \,e^{i(k\,z - \omega \,t)} + c.c.$$

where $\gamma$ signifies a wave train little amount.

When $\gamma$ has a nontrivial solution in Eq. (48), the dispersion relation grows to be nontrivial.

50 $$\omega^{2} + (\beta_{1} + i\beta_{2} )\omega + (\beta_{3} + i\beta_{4} ) = 0,$$

where $$\beta_{1} = b_{1} + k\,a_{4} ,$$$$\beta_{2} = - a_{1} ,$$$$\beta_{3} = - k\,b_{2} + a_{3} - k^{2} \,a_{5}$$ and $$\beta_{4} = k\,a_{2} + b_{3}$$.

A linear dispersion relation that distributes along the two cylindrical flowing fluids along with the fundamental axis is represented by Eq. (50). It contains of the wave number and growth rate that correspond to all of the problem physical constraints. According to the linear stability theory, the growth rate behavior determines whether a period of time is stable or unstable. If the imaginary part is positive, the disruption will spread with time. Consequently, the system progresses linear instability. The scheme would become linearly stable when the imaginary portion of is negative since it will cause a reduction in the disturbance. In these situations, the stability or instability depends on how the imaginary part of the system behaves and whether it oscillates for any time. It should be noted that the real part of the frequency of the surface wave has no implication in the stability configuration. This section major focus on examining the system stability under a linear approach. Furthermore, the nonlinear stability analysis has different criteria rather than the linear one. Simply, the linear modes analysis does not valid throughout the linear sense. At this point, the Routh-Hurwitz theory is applied to achieve the instability criteria of the dispersion relationship (50), see Peña^49^. Subsequently, the following are the stability requirements:

51 $$\beta_{1} > 0$$

and

52 $$\beta_{3} \beta_{1}^{2} + \beta_{1} \beta_{2} \beta_{4} - \beta_{4}^{2} > 0.$$

The analysis shows that $\beta_{1}$ is independent of the electric strength $E_{0}^{2}$. Typically, the stability profile needs to display $E_{0}^{2}$ against the wave numeral. To verify the implication of condition (51), all the character values should satisfy this condition, in order that this condition can become automatically satisfied. On the other hand, the condition (52) can be arranged to be formulated in the following form:

53 $$\Gamma \,E_{0}^{2} + {\rm K} > 0,$$

where $\Gamma = - k\,b_{21} \,\beta_{2}^{2}$ and $K = \beta_{1}^{2} ( - k\,b_{22} + a_{3} - k^{2} a_{5} ) + \beta_{1} \beta_{2} \beta_{4} - \beta_{4}^{2}$, the coefficients $b{}_{21}\,$ and $b_{22}$ are known from the context.

Before performing the numerical calculation, the stability requirement given by inequalities (51) and (53) can be represented in satisfactory formulae. To this end, they must be written in a dimensionless arrangement. This can be prepared in a number of numerous techniques, contingent on the standards selected for distance and duration. These characteristics may be chosen as follows: distance and duration attributes can be chosen as $R$ and $\sqrt {\rho_{l} R^{3} /T}$, respectively. The other dimensionless quantities can be stated as follows:

$$\rho _{g} = \rho ^{*} \,\rho _{l} ,\varepsilon _{g} = \varepsilon \,^{*} \varepsilon _{l} ,V_{g} = V^{*} \,V_{l} ,\mu _{g} = \mu ^{*} \,\mu _{l} ,\,\mu ^{\prime}_{g} = \mu ^{\prime*} \,\mu ^{\prime}_{l} ,k = k^{ * } /R,\,\,\lambda _{2} = \lambda ^{*} \lambda _{1} \,\,{\text{and}}\,\,\,\,E_{0}^{2} = E_{0}^{{*2}} T/R,$$

The procedure in considering the previous choice yields the following dimensionless numbers in the dispersion relationship:

**Table:** 

Non-Dimension Physical Number	Mathematical Formal
Darcy number $D_{a}$	$\kappa^{2} /R^{2}$
Weber number $W_{e}$	$\rho_{l} V_{l}^{2} R/T$
Laplace number $L_{a}$	$TR\rho_{l} /\mu_{l}^{2}$
Reynolds number $R_{e}$	$\rho_{l} V_{l} R/\mu_{l}$
Elasticity number $E_{l}$	$\lambda_{l} \mu_{l} /R^{2} \rho_{l}$
linear MHT dimensionless constant $\alpha_{1}^{*}$	$R^{2} V_{l} \alpha_{1} /T$
Electric Bond number $E_{0}^{*2}$	$E_{0}^{2} R\,\varepsilon_{l} /T$

where $- \,*\,$ describes the dimensionless amounts, which desired to be dropped later for a sake of easiness.

**Figure 2:**
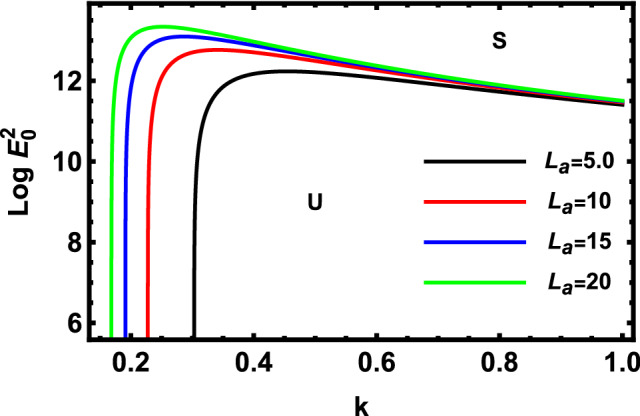
Displays the deviation of the growing amount against the wave numeral of numerous standards for the Laplace Numeral $L_{a}$.

**Figure 3:**
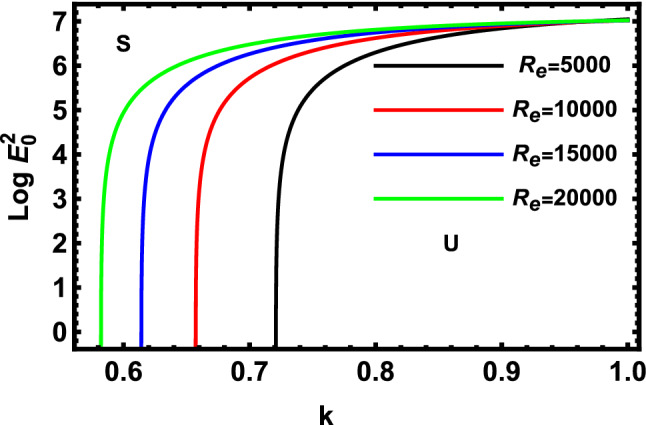
Displays the deviation of the growing amount against the wave numeral of numerous standards for the Reynolds Numeral $R_{e}$.

**Figure 4:**
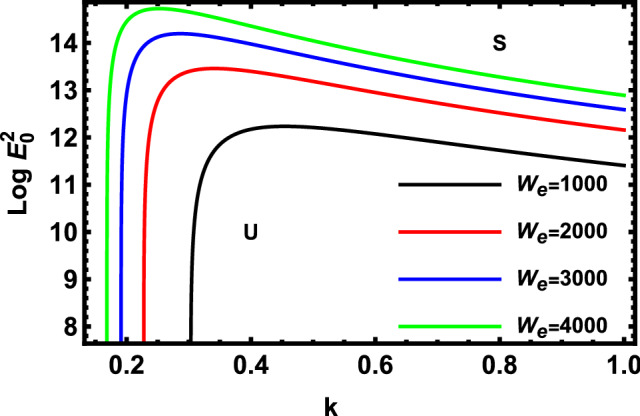
Displays the deviation of the growing amount against the wave numeral of numerous standards for the Weber Numeral $W_{e}$.

**Figure 5:**
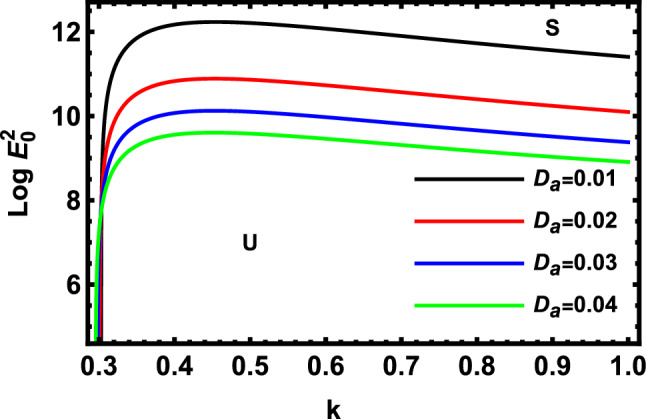
Displays the deviation of the growing amount against the wave numeral of numerous standards for the Darcy Numeral $D_{a}$.

**Figure 6:**
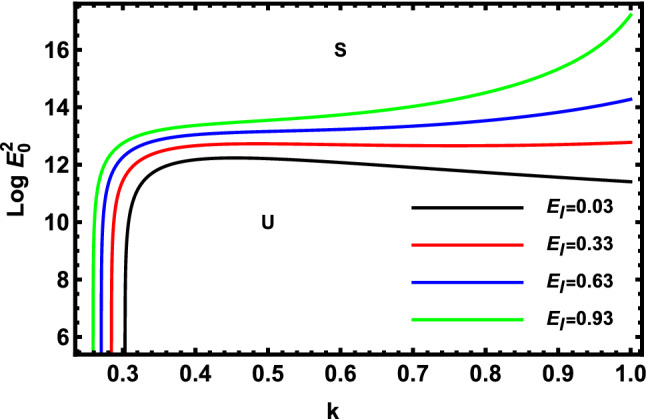
Displays the deviation of the rising amount against the wave numeral of numerous standards for the Elasticity Numeral $E_{l}$.

**Figure 7:**
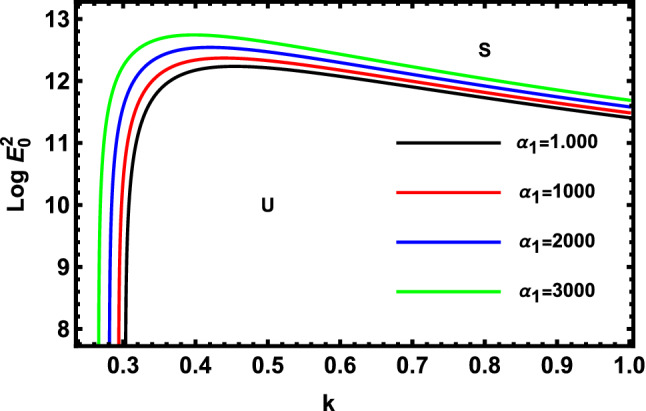
Displays the deviation of the growing amount against the wave numeral of numerous standards for the MHT constant $\alpha_{1}$.

As previously stated in inequity (51) should be satisfied. Accordingly, all the following graphs are drawn in a specific domain, where the criterion (51) is automatically satisfied. Furthermore, the calculation revealed that the factor $\Gamma$ is always positive. This indicates that the longitudinal unchanged electric field always provides a stabilizing influence, which is a preliminary conclusion. It is established by the earlier works of Melcher and Taylor^1^, Saville^2^, and many other references cited herein. At this argument, our focus is totally based on the criterion (53). Regularly, the electric field strength $Log\,E_{0}^{2}$ is often plotted versus the wave numeral $k$. Actually, the controlling $Log\,E_{0}^{2}$ instead of $\,E_{0}^{2}$ permits us to accumulate larger and smaller amounts at the equal vertical axis scale. It also produces smoothness of the sharp curves. In what follows, the letter $S$ signifies the stable zones. Concurrently, the symbol $U$ represents the unstable zones. Actually, the dimensionless procedure allows us to choose the data that has been given before, refraining from real physical problems. Consequently, the Figs. 2, 3, 4, 5, 6 and 7 depict a sample selection structure having the subsequent characteristics:

$$V = 0.05,\,\rho = 1,\,R_{e} = 5,\,L_{a} = 5,\mu = 1,\,\,\varepsilon = 0.1,\,W_{e} = 1000,\,\alpha = 1,\,\lambda = 0.5\,\,{\text{and}}\,{\text{ D}}_{{\text{a}}} = 0.01.$$

Figure 2 displays the deviation of the logarithm of the dimensionless electric strength $Log\,E_{0}^{2}$ against the dimensionless wave numeral $k$ for various values of the Laplace numeral $L_{a}$. Physically, the Laplace numeral is employed to describe the free-surface fluid dynamics. It is the proportion of surface tension to momentum transfer inside a liquid. The figure shows the implication of the Laplace numeral $L_{a}$. It is clear that the stable zone rises as the values $L_{a}$ are reduced, which demonstrates a destabilizing impact of this factor. Consequently, the Laplace numeral has a destabilizing influence on the structure. Physically, the growth in the interfacial tension, or the undisturbed cylindrical radius, or density causes more destabilization effects on the non-Newtonian cylindrical fluid. As a conclusion, for the reasonable assumptions, the non-Newtonian fluid jet is destabilized further at large amounts of the fluid Laplace numeral. Additionally, it is discovered that viscosity inhibits the emergence and growth of non-Newtonian fluid jet short-wave instability.

Figure 3 demonstrates the deviation of the logarithm of the dimensionless electric strength $Log\,E_{0}^{2}$ vs the non-dimensional wave numeral $k$ of various values of the Reynolds numeral $R_{e}$. As known, the Reynolds numeral aids in the calculation of flow forms in various liquid flow circumstances. The figure indicates the consequence of Reynolds numeral $R_{e}$, which is represented as $R_{e}^{2} = W{}_{e}\,L_{a} ,$ considering that Weber number has a constant value equal to $1000$, and that the values of the Laplace numeral are changed as shown in Fig. 2. It is apparent that the stable zone increases as the values of $R_{e}$ are reduced. Therefore, the Reynolds numeral provides a destabilizing impact on the considered model. Physically, the rise in the streaming fluid cylinder destabilizes the non-Newtonian fluid jet. It is established that the non-Newtonian fluid jet is destabilized further clearly at great values of the Reynolds numeral for the supposed situations. Furthermore, the viscosity prevents the onset and development of short-wave stability of non-Newtonian fluid jets. Subsequently, a comparable finding was verified^50^. It should be noted that the Reynolds numeral $R_{e}$ helps predict flow patterns in different fluid flow situations by measuring the ratio between inertial and viscous forces. At low Reynolds numbers, flows tend to be dominated by laminar (sheet-like) flow, while at high Reynolds numbers flows tend to be turbulent. The turbulence results from differences in the fluid speed and direction, which may sometimes intersect or even move counter to the overall direction of the flow (eddy currents). These eddy currents begin to churn the flow, using up energy in the process, which for liquids increases the chances of cavitation. The Reynolds number has wide applications, ranging from liquid flow in a pipe to the passage of air over an aircraft wing. It is used to predict the transition from laminar to turbulent flow and is used in the scaling of similar but different-sized flow situations, such as between an aircraft model in a wind tunnel and the full-size version. The predictions of the onset of turbulence and the ability to calculate scaling effects can be used to help predict fluid behavior on a larger scale, such as in local or global air or A large Reynolds number indicates that viscous forces are not important at large scales of the flow. With a strong prevalence of inertial forces over viscous forces, the largest scales of fluid motion become unsuppressed and there is not enough viscosity to dissipate their motions. Since the Lorentz force is always perpendicular to the flow velocity and it impedes movement when the flow becomes turbulent (at high Reynolds numbers), the Lorentz force becomes not effective to dampen the flow, and hence the flow is destabilized as shown.

Figure 4 demonstrates the deviation of the logarithm of the dimensionless electric strength $Log\,E_{0}^{2}$ vs the non-dimensional wave numeral $k$ of various values of Weber number $W_{e}$. Physically, the Weber numeral is frequently employed in the analysis of fluid flows involving two separate liquids, especially multi-phase flows through sharply rounded surfaces. It is a measure of the fluid inertia with respect to its interfacial tension. The figure can assist in understanding how a thin film flows and how droplets and bubbles improve. As a conclusion, this picture demonstrates that $W_{e}$ portrays a destabilizing impact on the given structure. Basically, the rise of this factor means that the primary flowing also increases. As known, the destabilizing impact of KHI is an early finding confirmed by several authors cited here. Especially, this finding accords with those early obtained^50^. This depicts how amplifying the velocity, radius, or density of non-Newtonian liquid jets causes them to become unstable. Additionally, it is implied that the non-Newtonian liquid jet destabilizes more quickly with larger liquid Weber numbers. It should be noted that the Weber number $W_{e}$ is a dimensionless number in fluid mechanics that is often useful in analyzing fluid flows where there is an interface between two different fluids, especially for multiphase flows with strongly curved surfaces. It can be thought of as a measure of the relative importance of the fluid inertia compared to its surface tension. The quantity is useful in analyzing thin film flows and the formation of droplets and bubbles.

Figure 5 indicates the deviation of the logarithm of the dimensionless electric strength $Log\,E_{0}^{2}$ against the non-dimensional wave number $k$ of various of Darcy number. Physically, the Darcy number is important when discussing heat conduction through porous materials such as powdered metals, foams, ceramics, etc. The Darcy number in fluid dynamics due to porous media typically demonstrates the relative influence of the medium permeability versus its cross-sectional area when the diameter is squared. The Darcy number also has a considerable impact on the entropy generation caused by convection inside porous triangular additions. This chart research reveals that the unstable areas also experience. This result shows that the stable zone rises as the values $D_{a}$ rise, too. As shown from the mathematical formula $D_{a}$, the growth in $D_{a}$ produces an increase in the permeability of the permeable medium. This leads to a reduction in the influence of porous media. This outcome is supported with the results of previous studies^51^. Simultaneously, the increase of $D_{a}$ means a decrease in the radius of the cylinder. As shown by several researchers, the increase in the cylindrical radius leads to a stabilizing influence as previously shown by Moatimid et al.^41^.

Figure 6 demonstrates the deviation of the logarithm of the dimensionless electric strength $Log\,E_{0}^{2}$ against the dimensionless wave numeral $k$ of various values of Elasticity numeral $E_{l}$. Subsequently, one concludes that the elasticity numeral has a destabilizing influence on the approach that is being investigated. Physically, the increase of the relaxation duration or viscosity generates that the non-Newtonian cylindrical fluid to be more unstable. Therefore, it is concluded that, as given in the stated circumstances, the non-Newtonian fluid jet is further clearly destabilized at high values of the fluid Elasticity numeral. Furthermore, the effects of radius, or density are found to stand in contrast to the findings yielded in^52^.

Figure 7 demonstrates the deviation of the logarithm of the dimensionless electric strength $Log\,E_{0}^{2}$ against the dimensionless wave numeral $k$ of various values of the linear MHT dimensionless constant. Consequently, one concludes that $\alpha_{1}$ has a destabilizing effect on the methodology that is being inspected. Physically, the increase in the undisturbed cylindrical radius, velocity, and MHT constant forces the non-Newtonian cylindrical fluid to be more unstable. Additionally, more heat is transferred to a point at the trough and less heat is prevented from the site as MHT increases across the interface. Therefore, it is stated in the given circumstances that the non-Newtonian fluid jet is further clearly destabilized at high values of the fluid MHT dimensionless constant. Furthermore, the interfacial tension of the non-Newtonian liquid jet is observed to avoid the beginning and improvement of short-wave stability as earlier obtained^53^.

To this end, the linear stability approach has been finished. Therefore, the next Section is depicted to display the stability configuration throughout a nonlinear approach. This approach seeks a nonlinear partial differential equation that governs the interface displacement. Fundamentally, the first step concerns with the derivation of a Ginzburg–Landau equation, which regulates the nonlinear instability standards. Consequently, the theoretical outcomes will interpret the transition curves, which classified the stability/instability zones. Apart from the linear methodology, for more accuracy, the nonlinear criteria will reveal different transition curves.

## Nonlinear stability methodology

Returning to the aforementioned nonlinear characteristic equation as given in Eq. (47), the interface displacement nonlinear characteristic equation is assessed up to the third order of $\eta$ to give

54 $$D(k,\omega )\eta = \alpha (k,\omega )\eta^{2} + \beta (k,\omega )\eta^{3}$$

where $\alpha \left( {\omega ,\,k} \right)$ and $\beta \left( {\omega ,\,k} \right)$ are the second-and third-order nonlinear constants, correspondingly, and $D\left( {\omega ,\,k} \right)$ is the linear coefficient.

The various time scale techniques will be used to build the analysis that follows. This methodology is used in order to demonstrate a problem-solving strategy as a function of two or additional independent parameters. The ratio of a perfect wavelength, periodic time, or modulation time scale can therefore be assumed to be a small parameter. The independent parameters $z$ and $t$ may be extended to include more independent parameters in the manner shown below.

55 $$Z_{n} = \zeta^{n} z\;\;\;{\text{and}}\;\;\;T_{n} = \zeta^{n} t\;\;\;\;n = 0,1,2,........$$

while $Z_{0}$ and $T_{0}$ are both valid examples of fast variations, the slow ones are $Z_{1} ,\,\,T_{1} ,\,Z_{2} ,\,\,{\text{and}}\,\,T_{2}$. The following derivative expansions can be used to express the differential operators:

56 $$\frac{\partial }{\partial z} \equiv k\frac{\partial }{\partial \theta } + \zeta \frac{\partial }{{\partial Z_{1} }} + \zeta^{2} \frac{\partial }{{\partial Z_{2} }} + ........,{\text{and}}\;\;\frac{\partial }{\partial t} \equiv - \omega \frac{\partial }{\partial \theta } + \zeta \frac{\partial }{{\partial T_{1} }} + \zeta^{2} \frac{\partial }{{\partial T_{2} }} + ........$$

when $\theta = kZ_{0} - \omega T_{0}$, the lowest order is indicated.

The partial derivatives of time and space linear operation are

57 $$M\left( {\frac{\partial }{\partial z},\frac{\partial }{\partial t}} \right)\eta = 0.$$

The operator $M$ then develops

58 $$M\left( {(ik, - i\omega ),\,\zeta \left[ {\frac{\partial }{{\partial Z_{1} }},\frac{\partial }{{\partial T_{1} }}} \right],\,\,\,\,\zeta^{2} \left[ {\frac{\partial }{{\partial Z_{2} }},\frac{\partial }{{\partial T_{2} }}} \right],........} \right) = 0.$$

The operator manifestation M may be expanded in like powers of $\zeta$. By constructing Taylor's expansion about $(ik, - i\omega )$ up to $O(\zeta^{2} )$, this may be achieved. Consequently, one may get

59 $$M \to M_{0} + \zeta \,M_{1} + \zeta^{2} M_{2} + .....,$$

where

60 $$M_{0} \equiv (k, - \omega )\frac{\partial }{\partial \theta },$$

61 $$M_{1} \equiv i\left( {\frac{{\partial M_{0} }}{\partial \omega }} \right)\frac{\partial }{{\partial T_{1} }} - i\left( {\frac{{\partial M_{0} }}{\partial k}} \right)\frac{\partial }{{\partial Z_{1} }}$$

and

62 $$M_{2} \equiv i\left( {\frac{{\partial M_{0} }}{\partial \omega }} \right)\frac{\partial }{{\partial T_{2} }} - i\left( {\frac{{\partial M_{0} }}{\partial k}} \right)\frac{\partial }{{\partial Z_{2} }} - \frac{1}{2}\left( {\frac{{\partial^{2} M_{0} }}{{\partial \omega^{2} }}} \right)\frac{{\partial^{2} }}{{\partial T_{1}^{2} }} - \frac{1}{2}\left( {\frac{{\partial^{2} M_{0} }}{{\partial k^{2} }}} \right)\frac{{\partial^{2} }}{{\partial Z_{1}^{2} }} + \left( {\frac{{\partial^{2} M_{0} }}{\partial k\,\partial \omega }} \right)\frac{{\partial^{2} }}{{\partial Z_{1} \partial T_{1} }}.$$

Putting the development of the operator (59) into Eq. (57) and taking Eq. (49) into consideration, one realizes

63 $$\left( {D_{0} + \delta \,D_{1} + \delta^{2} D_{2} } \right)\gamma = 0,$$

where the linear dispersion connection is described by $D_{0} \equiv 0$,

64 $$D_{1} \equiv i\left( {\frac{\partial D}{{\partial \omega }}} \right)\frac{\partial }{{\partial T_{1} }} - i\left( {\frac{\partial D}{{\partial k}}} \right)\frac{\partial }{{\partial Z_{1} }}$$

and

65 $$D_{2} \equiv i\left( {\frac{\partial D}{{\partial \omega }}} \right)\frac{\partial }{{\partial T_{2} }} - i\left( {\frac{\partial D}{{\partial k}}} \right)\frac{\partial }{{\partial Z_{2} }} - \frac{1}{2}\left( {\frac{{\partial^{2} D}}{{\partial \omega^{2} }}} \right)\frac{{\partial^{2} }}{{\partial T_{1}^{2} }} - \frac{1}{2}\left( {\frac{{\partial^{2} D}}{{\partial k^{2} }}} \right)\frac{{\partial^{2} }}{{\partial Z_{1}^{2} }} + \left( {\frac{{\partial^{2} D}}{\partial k\,\partial \omega }} \right)\frac{{\partial^{2} }}{{\partial Z_{1} \partial T_{1} }}.$$

Therefore, the nonlinearity of $\eta$ in parts of $\zeta$ can be expressed as follows:

66 $$\eta = \sum\limits_{n = 1}^{3} {\zeta^{n} } \eta_{n} \left( {\theta ,Z_{1} ,Z_{2} ,T_{1} ,T_{2} } \right) + O\left( {\zeta^{4} } \right).$$

Associating the coefficients of the identical exponents of $\zeta$ on both sides after combining Eqs. (54), (59), and (66), one can determine the orders in $\zeta$ as follows:

67 $$\zeta :\;\;\;\;\;\;\;\;\;\;\;\;\;\;\;\;\;\;\;\;\;\;\;\;\;\;\;\;\;\;\;\;\;\;\;\;\;\;\;\;\;\,M_{0} \eta_{1} = 0\,\,,$$

68 $$\zeta^{2} :\;\;\;\;\;\;\;\;\;\;\;\;\;\;\;\;\;\;\;\;\;\;\;\;\;\;\;\;\;\;\;\;\;M_{0} \eta_{2} = - M_{1} \eta_{1} + \alpha \,\eta_{1}^{2} ,$$

and

69 $$\zeta^{3} :\,\;\;\;\;\;\;\;\;\;\;\;\;\;\;\;\;\;\;\;\;\;\;\;\;\;\;\;\;\;\;\;\;\;\;\;\;M_{0} \eta_{3} = - M_{1} \eta_{2} - M_{2} \eta_{1} + 2\alpha \,\eta_{1} \,\eta_{2} + \beta \eta_{1}^{3}$$

One may presume the following quasi-monochromatic wave solution as the lowest order approximation of

70 $$\eta_{1} = \gamma (Z_{1} ,Z_{2} ,T_{1} ,T_{2} )\,\,e^{i\theta } + c.c.$$

Equation (68) changes to

71 $$M_{0} \eta_{2} = - i\left[ {\left( {\frac{\partial D}{{\partial k}}} \right)\frac{\partial \gamma }{{\partial Z_{1} }} - \left( {\frac{\partial D}{{\partial \omega }}} \right)\frac{\partial \gamma }{{\partial T_{1} }}} \right]\,\,e^{i\theta } + \alpha \,\left( {\gamma^{2} e^{2i\theta } + 2\gamma \,\overline{\gamma } } \right) + c.c.,$$

where $\overline{\gamma }$ signifies the complex conjugate of $\gamma$.

The secular terms in Eq. (71) correspond to the coefficient of $e^{i\theta }$. The following solvability criterion results from the term elimination:

72 $$\left( {\frac{\partial D}{{\partial k}}} \right)\frac{\partial \gamma }{{\partial Z_{1} }} - \left( {\frac{\partial D}{{\partial \omega }}} \right)\frac{\partial \gamma }{{\partial T_{1} }} = 0,$$

It produces a complicated relationship. A uniformly valid expansion of the function $\eta_{2}$ takes the following form thanks to this solvability condition:

73 $$\eta_{2} = \frac{\alpha }{\Omega }\gamma^{2} e^{2i\theta } + c.c.\,\,.$$

By replacing both the parameters $\omega$ and $k$ with $2\omega$ and $2k$, correspondingly, the linear dispersion function $D\left( {\omega ,k} \right)$ can be used to structure the non-zero denominator $\Omega$. It must be mentioned that the disappearance of $D\left( {2\omega ,2k} \right)$ produces the well-known resonance curve if $D\left( {2\omega ,2k} \right)$ is a real function. Overall, the harmonic resonance can happen if $\left( {k,\,\omega } \right)$ and $\left( {nk,\,n\omega } \right)$ meet the similar dispersion relation when $n\,$ is a positive integer; for example, see Nayfeh^54^. Subsequently, the denominator transforms into a true positive function that cannot disappear. Subsequently, as will be seen later, a Ginzburg–Landau equation will be obtained.

The uniformly third order solution, which is obtained by combining Eqs. (69), (70) and (73), advances to the following solvability condition.

74 $$i\left( {\frac{\partial D}{{\partial \omega }}} \right)\frac{\partial \gamma }{{\partial T_{2} }} - i\left( {\frac{\partial D}{{\partial k}}} \right)\frac{\partial \gamma }{{\partial Z_{2} }} - \frac{1}{2}\left( {\frac{{\partial^{2} D}}{{\partial \omega^{2} }}} \right)\frac{{\partial^{2} \gamma }}{{\partial T_{1}^{2} }} - \frac{1}{2}\left( {\frac{{\partial^{2} D}}{{\partial k^{2} }}} \right)\frac{{\partial^{2} \gamma }}{{\partial Z_{1}^{2} }} + \left( {\frac{{\partial^{2} D}}{\partial k\,\partial \omega }} \right)\frac{{\partial^{2} \gamma }}{{\partial Z_{1} \partial T_{1} }} = \left( {2\frac{{\alpha^{2} }}{\Omega } + 3\beta } \right)\gamma^{2} \overline{\gamma } .$$

The second and third-order issues can then be solved in order to obtain the equation amplitude evolution. The non-security circumstances in the existence of the regularly applicable solutions in the second order, as given by Eq. (72), might be stated by using the method created^54^.

75 $$\frac{\partial \gamma }{{\partial T_{1} }} + V_{g} \frac{\partial \gamma }{{\partial Z_{1} }} = 0\,\,,\,\,\,\,\,{\text{Provided}}\,\,{\text{that}}\,\,\,\,\,\,\frac{\partial D}{{\partial \omega }} \ne 0,$$

where $V_{g} = - \frac{\partial D}{{\partial \,k}}\left( {\frac{\partial D}{{\partial \omega }}} \right)^{ - 1}$ is the wave train solution's group velocity.

According to Eq. (75), the wave approximates a second order group velocity. This indicates that the slow parameters $Z_{1} ,\,T_{1}$ through the mixture $(Z_{1} - V_{g} T_{1} )$ affect the amplitude $\gamma$. One must arrive at the third-order equation given by Eq. in order to improve the amplitude modulation for the increasing waves (63). A single equation can be created by simplifying and combining the solvability criteria (72) and (74). The derivatives in $\gamma$ can be removed from Eq. (75) by using Eq. (74). Consequently, one may write

76 $$\frac{{\partial^{2} \gamma }}{{\partial Z_{1} \partial T_{1} }} = - V_{g} \frac{{\partial^{2} \gamma }}{{\partial Z_{1}^{2} }}\,\,,\,\,\,\,\,\,{\text{and}}\,\,\,\,\,\,\,\,\frac{{\partial^{2} \gamma }}{{\partial T_{1}^{2} }} = V_{g}^{2} \frac{{\partial^{2} \gamma }}{{\partial Z_{1}^{2} }}.$$

When Eq. (76) is substituted into Eq. (74), Eq. (55) is divided by $\left( {\frac{\partial D}{{\partial \omega }}} \right)$ and used to get the result.

77 $$i\left( {\frac{\partial \gamma }{{\partial t}} + V_{g} \frac{\partial \gamma }{{\partial z}}} \right) + P\frac{{\partial^{2} \gamma }}{{\partial z^{2} }} = \zeta^{2} Q\,\gamma^{2} \overline{\gamma } ,$$

where $P$ and $Q$ are

$$P = - \frac{1}{2}\left( {\frac{\partial D}{{\partial \omega }}} \right)^{ - 1} \left( {V_{g}^{2} \frac{{\partial^{2} D}}{{\partial \omega^{2} }} + 2V_{g} \frac{{\partial^{2} D}}{\partial \omega \,\partial k} + \frac{{\partial^{2} D}}{{\partial k^{2} }}} \right),\;\;\;\;\;{\text{and}}\;\;\;Q = \left( {\frac{\partial D}{{\partial \omega }}} \right)^{ - 1} \left( {\frac{2\alpha }{\Omega } + 3\beta } \right)\,.$$

It is clear that $P$ and $Q$ are complex constants.

Elhefnawy^55^ demonstrated the Grander-Morikawa alteration, which results in

78 $$\,\,\,\varsigma = \zeta (z - V_{g} t),\,\,\,\,\,\,\,\,\,\,\,\,\,\,\,\,\,\,\,\,\,\,\,\,\,\,\,\,\,\,\,\,\,{\text{and}}\,\,\,\,\,\,\,\,\,\,\,\,\,\,\,\,\,\,\,\,\,\,\,\,\,\,\,\,\,\,\,\tau = \zeta^{2} t,$$

Then, Eq. (78) is reduced to

79 $$i\frac{\partial \gamma }{{\partial \tau }} + P\frac{{\partial^{2} \gamma }}{{\partial \varsigma^{2} }} = Q\,\gamma^{2} \overline{\gamma } ,$$

The well-known Ginzburg–Landau equation is (79). The complex constants $P$ and $Q$ mean that

80 $$P = P_{r} + i\,P_{i} \,\,\,\,\,\,\,\,\,\,\,\,\,\,\,\,\,\,\,\,\,\,\,{\text{and}}\,\,\,\,\,\,\,\,\,\,\,\,\,\,\,\,Q = Q_{r} + i\,Q_{i} ,$$

It is obvious that the characteristics of factors $P$ and $Q$ result from the characteristics of the constants of the nonlinear distinguishing Eq. (54). Now, Eq. (79) is comparable to the equation previously found by Elhefnawy^55^. The stability conditions of Ginzburg–Landau Eq. (79) have been discovered by Lang and Newell^56^. They established that the linear perturbation is stable under the following restrictions

81 $$Q_{i} < 0,\,\,$$

and

82 $$P_{r} Q_{r} + P_{i} Q_{i} > 0.$$

Otherwise, the system becomes unstable. The well-known Schrödinger equation results as a particular case when the imaginary components in the aforementioned requirements are taken out. Nayfeh^56^ derived the stability of Schrödinger equation.

The transition curves as given by the equalities of (81) and (82) should be addressed in a practical dimensionless form prior to dispersing the numerical calculations. This can be made in a variety of approaches in light of the choice of time, length, and mass appearances. Consider that the values of the factors $1/\omega$,$a$, and $T/\omega^{2}$, respectively, describe the properties of time, length, and mass.

After complex but basic calculations, the transition curve provided by $Q_{i} = 0$ may be organized in a third-degree polynomial of $E_{0}^{2}$ as follows.

83 $$Y_{3} (E_{0}^{2} )^{3} + Y_{2} (E_{0}^{2} )^{2} + Y_{1} E_{0}^{2} + Y_{0} = 0.$$

However, the fifth-degree polynomial on $E_{0}^{2}$ can be used to arrange the second transition curve as given by $P_{r} Q_{r} + P_{i} Q_{i} = 0$ as follows:

84 $$G_{5} (E_{0}^{2} )^{5} + G_{4} (E_{0}^{2} )^{4} + G_{3} (E_{0}^{2} )^{3} + G_{2} (E_{0}^{2} )^{2} + G_{1} E_{0}^{2} + G_{0} = 0.$$

where the constants provided in Eqs. (83) and (84) are understood from the context. They will be crossed out in order to make the paper shorter.

The transition curves provided in Eqs. (83) and (84) will be investigated in order to demonstrate the stability standards used during this nonlinear stability methodology.

For this objective, consider the following particular system:

$$\lambda_{1} = 0.2,\;\lambda_{2} = 0.5,\;\mu_{l} = 0.3,\;\mu_{g} = 0.4,\;\rho_{l} = 0.1,\;\rho_{g} = 0.5,\;v_{l} = 0.5,\;v_{g} = 0.1,\;\alpha_{1} = 0.2,\;\alpha_{2} = 6,\;\alpha_{3} = 0.4,$$

$$b = 4,\;R = 2,\;\kappa = 0.01,\;\varepsilon_{l} = 3,\;{\text{and}}\;\varepsilon_{g} = 0.2.$$

**Figure 8:**
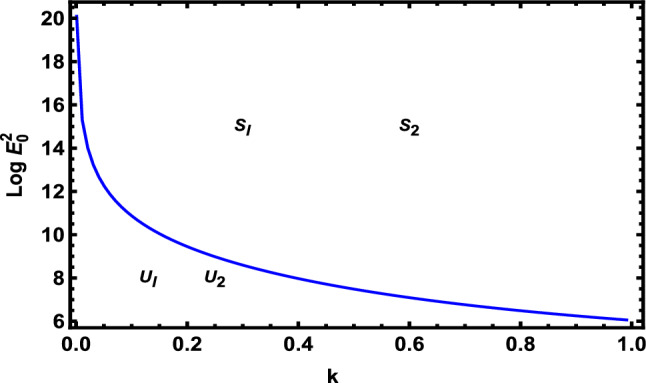
Deviation of $\log E_{0}^{2}$ with $k$ to illustrate the involvement of Eqs. (83) and (84).

In light of the above data, the calculations revealed that only one positive real root is produced by Eq. (83), however, the other two roots are complex conjugates. Really, this comes from algebra. Additionally, Eq. (84) generates only one positive real root and the other four roots in Eq. (84) are complex conjugates. Therefore, the two criteria revealed only two transition curves. Actually, the other sixth curves have no implication on the stability profile. As a surprise, the only two transition curves are coincident with each other. The symbols $S_{1} ,\,\,S_{2}$ indicate that the two inequalities (81) and (82) are automatically satisfied. By contrast, symbols $U_{1} ,\,\,U_{2}$ mean that the above stability criteria are not satisfied. Unfortunately, the nonlinear theory yields one transition curve like the linear theory. The inspection of these transition curves shows that the transition curve in the linear sense behaves like an increasing function. On the other hand, the transition curve throughout the nonlinear theory reveals a decreasing function. Therefore, one can say that the stability zone in the nonlinear sense is larger than the linear approach. Consequently, in Fig. 8, $\log E_{0}^{2}$ is planned against the numeral of surface wavenumber $k$.

**Figure 9:**
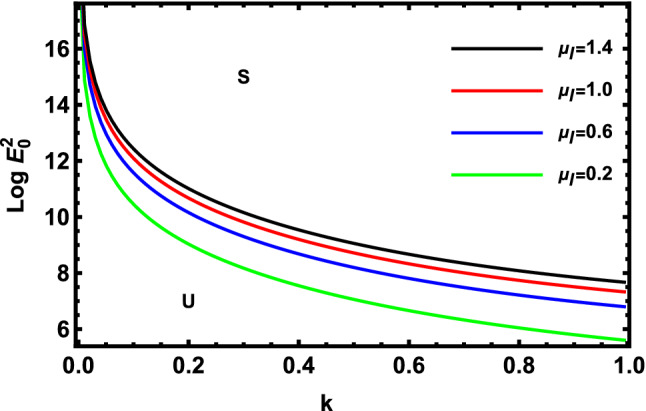
Displays the deviation of the growing amount versus the wave number of numerous standards for viscosities of liquid $\mu_{l}$.

Figure 9 demonstrates the deviation of the logarithm of the dimensionless electric strength $Log\,E_{0}^{2}$ vs the non-dimensional wave number $k$ of various values of viscosities of liquid $\mu_{l}$ in the nonlinear stability picture. From the examination of this picture, one concludes that the viscosity of liquid has a destabilizing impact on the approach that is being investigated. Physically, fluid resistance rises with linear viscosity and flow velocity is thus reduced, where viscosity is recognized as a factor in flow resistance. The internal fluid friction is displayed. A fluid with a high viscosity can resist motion because of the tremendous internal friction produced by its molecules. Chemically, when the intermolecular forces of attraction are strong, a material has a high viscosity. The easiest way to describe this phenomenon is to imagine two liquids rushing down a surface. This behavior is consistent with the Elasticity Number influence on the linear study previously seen in Fig. 6, where the Elasticity number is directly proportionate to the liquid viscosity. The finding is consistent with that which was previously achieved^58^.

**Figure 10:**
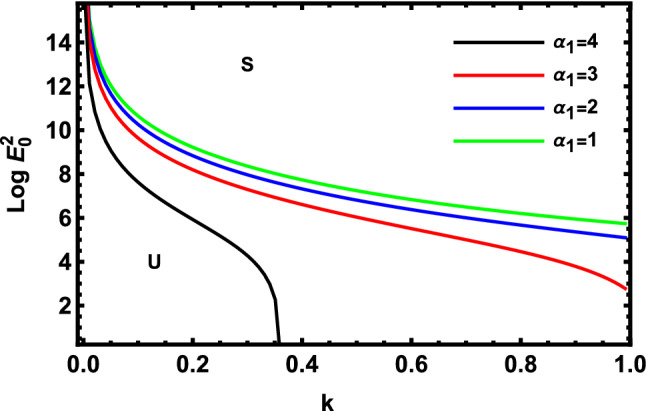
Displays the deviation of the growing amount versus the wave number of MHT constant $\alpha_{1}$.

**Figure 11:**
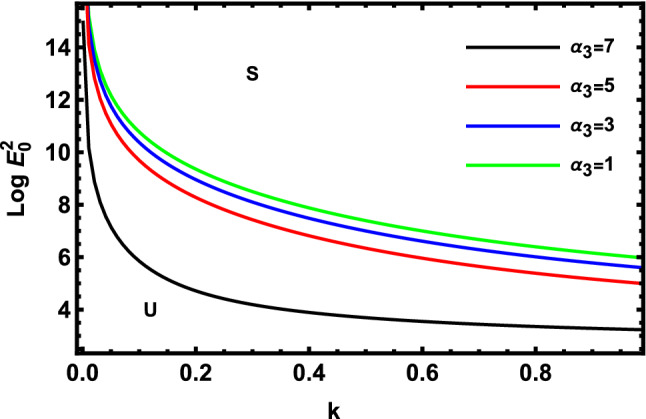
Displays the deviation of the growing amount versus the wave number of MHT constant $\alpha_{3}$.

The effects of MHT parameters $\alpha_{1}$ and $\alpha_{3}$, respectively, on the stability image are shown in Figs. 10 and 11. Figure 10 demonstrates the fluctuation of the logarithm of the dimensionless electric intensity $Log\,E_{0}^{2}$ against the dimensionless wave numeral $k$.Consequently, $\alpha_{1}$ has a stabilizing effect, as found in Fig. 10. The linear stability technique shown in Fig. 7 is incompatible with this influence. As seen in Fig. 11, $\alpha_{3}$ has a stabilizing impact. Thus, it is demonstrated that the movement of heat and mass has a stabilizing effect. Additional heat may be transmitted to a place where more heat is far from the position due to an increase in heat and mass transfer across the interface. As a result, it is possible to assert that the MHT factor plays a dual function in the stability standard. These findings coincide with those previously obtained by Sharma^18^.

**Figure 12:**
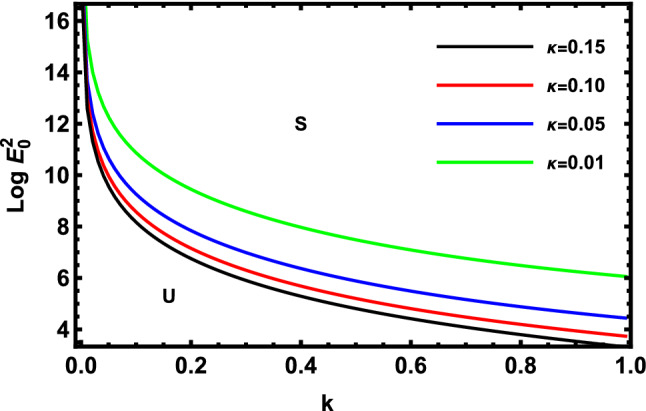
Displays the deviation of the growing amount versus the wave number of permeability porosity $\kappa$.

Figure 12 shows how the logarithm of the dimensionless electric field strength $Log\,E_{0}^{2}$ vs the non-dimensional wave number $k$ with various values of permeability porosity $\kappa$ varies. As shown,$\kappa$ has a stabilizing effect. Physically, the porous medium permeability is a quality that quantifies the formation capacity and ability to convey fluids. Because it regulates the direction and flow rate of the reservoir fluids in the formation, the rock permeability, k, is a significant rock characteristic. This effect is consistent with the impact of the Darcy numeral in the linear study as in Fig. 5, where the Darcy coefficient is directly proportional to the permeability of porosity.

**Figure 13:**
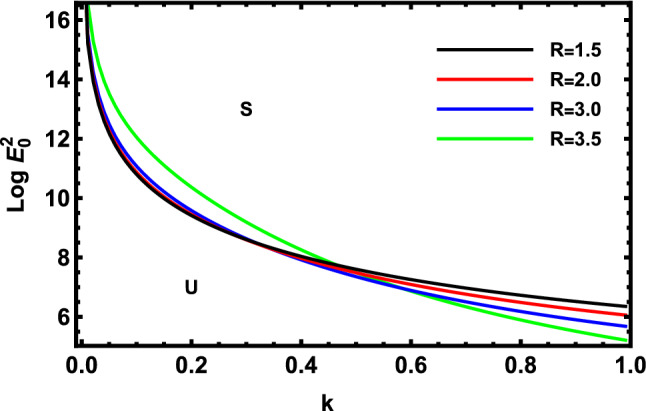
Displays the deviation of the growing amount versus the wave number of cylindrical undisturbed radius $R$.

Figure 13 shows how the logarithm of the dimensionless electric strength $Log\,E_{0}^{2}$ against the dimensionless wave numeral $k$ with various values of cylindrical undisturbed radius $R$ varies. As observed,$R$ has a dual role effect. We noted that for small values of wave numeral, the effect of radius is a destabilizing effect, whereas for larger values of wave numeral, the effect is changed to a stabilizing effect. This outcome is consistent with that which was previously achieved^8^.

## Estimated surface departure formulation

The goal in this Section is to arrive at a limited solution for the surface displacement. The nonlinear technique produces the nonlinear characteristic equation that is provided in Eq. (47), as was previously demonstrated. With the complex constants of the interface deviation $\eta (z,t)$, it forms a nonlinear second-order differential equation. Actually, this equation analysis in its current form is quite challenging. Physically, the amplitude departure $\eta (z,t)$ nature must be a real function. One may only consider the time dependent, i.e.,$\eta = \eta (0,t) = \Gamma (t)$, in order to make the subsequent calculations easier, the assumption is that

85 $$\eta (z,t) = \Gamma (t)\,\,e^{i\,k\,z} .$$

It will be easier to perform the calculations that follow if only the time dependence ($z = 0$), which is the true indicator of historical stability, is considered. It is possible to rewrite the nonlinear characteristic equation Eq. (47) as

86 $$\,\,\eta_{tt} \,(t) + \left( {a_{1} + i\,(b_{1} + ka_{4} )} \right)\,\,\eta_{t} + \left( {(a_{3} - kb_{2} - k^{2} a_{5} ) + i\,(ka_{2} + b_{3} )} \right)\,\,\eta + G(\eta ) = 0,$$

where $$G(\eta ) = \,\,\,i\,f_{0} \,\eta \, + \,\,\,(g_{1} + i\,f_{1} )\,\,\eta_{t} + \,(g_{2} + i\,f_{2} )\,\eta^{2} + \,(g_{3} + i\,f_{3} )\,\eta^{3} + \,(g_{4} + i\,f_{4} )\,\eta \,\,\eta_{t} \,\, + (g_{5} + i\,f_{5} )\,\eta^{2} \,\,\eta_{t} + g_{6} \eta \eta_{tt} + g_{7} \,\eta^{2} \,\eta_{tt}.$$

For this objectively, it is practical to consider the beginning circumstances listed below.:

87 $$\eta (0,0) = 0,\;{\text{and}}\;\eta_{t} (0,0) = \sum\limits_{j = 0}^{\infty } {\hbar^{j} \,\vartheta_{j} } ,$$

where the factors $\vartheta_{j}$ will be calculated later on after being combined with the initial features of the issue at hand. Disregarding the secular terms, this will be accomplished.

It follows that the Homotopy equation might be formulated as follows:

88 $$\eta_{tt} \,(t) + \tilde{\omega }^{2} \,\,\eta = - \,\,\hbar \left( {i\,(ka_{2} + b_{3} )\,\eta \, + \,\,\,\left( {a_{1} + i\,(b_{1} + ka_{4} )} \right)\,\eta_{t} + G(\eta )} \right)\,,\,\,\hbar \in [0,1]\,\,\,\,\,$$

where the symbol $\tilde{\omega }^{2} = (a_{3} - kb_{2} - k^{2} a_{5} )$ is the present model natural frequency. This method is primarily dependent on a small factor $\hbar$ and is based on an expanded frequency analysis. This method allows the enlarged artificial frequency $\varpi^{2}$ to be stated as follows^59^:

89 $$\varpi^{2} = \tilde{\omega }^{2} + \sum\limits_{j = 1}^{\infty } {\hbar^{j} \,\Re_{j} } .$$

The expanded form of the time-dependent function $\eta (0,t,\hbar )$ is as follows:

90 $$\eta (0,t,\hbar ) = \sum\limits_{j = 0}^{\infty } {\hbar^{j} \,\,\eta_{j} \,(t)\,} .$$

By combining Eqs. (89) and (88), the nonlinear characteristic equation can be rewritten as

91 $$\eta_{tt} + \varpi^{2} \,\,\eta = - \,\,\hbar \left( {i\,(ka_{2} + b_{3} )\,\eta - (\Re_{1} + \,\hbar \,\Re {}_{2}\, + \hbar^{2} \,\Re_{3} )\,\,\eta \,,\,\, + \,\left( {a_{1} + i\,(b_{1} + ka_{4} )} \right)\,\,\eta_{t} + G(\eta )} \right).$$

Considering the Laplace transform applied to Eq. (91) and the initial conditions specified in Eq. (87), the result is:

92 $$L_{T} \{ \,\eta (0,t,\hbar )\} = \frac{{\vartheta_{0} }}{{S^{2} + \varpi^{2} \,}} + \hbar \,\frac{{\vartheta_{1} }}{{S^{2} + \varpi^{2} \,}} + \hbar^{2} \,\frac{{\vartheta_{2} }}{{S^{2} + \varpi^{2} \,}} + ... + \frac{\hbar \,\,}{{S^{2} + \varpi^{2} \,}}L_{T} \{ (\Re_{1} + \,\hbar \,\Re {}_{2}\, + \hbar^{2} \,\Re_{3} )\,\,\eta + \,\,G(\eta )\,\} \,\,.\,$$

Employing the inverse Laplace transforms of both sides of Eq. (92), one finds

93 $$\eta (0,t,\hbar ) = \frac{{\vartheta_{0} }}{\varpi }\,\sin \,(\varpi \,t) + \hbar \,\frac{{\vartheta_{1} }}{\varpi }\,\sin \,(\varpi \,t) + \hbar^{2} \,\frac{{\vartheta_{2} }}{\varpi }\,\sin \,(\varpi \,t) + ... + L_{T}^{ - 1} \,\left[ {\frac{\hbar \,}{{S^{2} + \varpi^{2} \,}}} \right.L_{T} \left\{ {\,(\Re_{1} + \,\hbar \,\Re {}_{2}\, + \hbar^{2} \,\Re_{3} )\,\,\eta } \right.\, + \,\left. {\left. {G(\eta )\,} \right\}\,} \right]\,\,.$$

Utilizing Eq. (90) improvement the dependent parameter $\eta (0,t,\hbar )$ and then locating the constants with equal powers $\hbar$ on both sides, one is able to obtain

94 $$\hbar^{0} :\,\,\,\eta_{0} \,(0,t) = \frac{{\vartheta_{0} }}{\varpi }\,\sin \,\varpi \,t\,,$$

95 $$\begin{aligned} \hbar :\,\eta _{1} \,(0,t) = & \frac{{\vartheta _{1} }}{\varpi }\,\sin \,\varpi \,t + L_{T}^{{ - 1}} \,\left[ {\frac{1}{{S^{2} + \varpi ^{2} \,}}} \right.L_{T} \left\{ {\Re _{1} \,\eta _{0} \,(0,t)} \right.\, + \,\,i\,f_{0} \,\eta _{0} (0,t)\, + \,\,\,(g_{1} + i\,f_{1} )\,\,\eta _{{0t}} \,(0,t)\, \\ & + \,(g_{2} + i\,f_{2} )\,\eta _{0}^{2} (0,t) + (g_{3} + i\,f_{3} )\,\eta _{0}^{3} (0,t) + \,(g_{4} + i\,f_{4} )\,\eta _{0} (0,t)\,\,\eta _{{0t}} \,(0,t) \\ & + (g_{5} + i\,f_{5} )\,\eta _{0}^{2} (0,t)\,\,\eta _{{0t}} \,(0,t) + g_{6} \,\eta _{0} (0,t)\,\,\eta _{{0tt}} \,(0,t) + \left. {\left. {g_{7} \,\eta ^{2} _{0} (0,t)\,\,\eta _{{0tt}} \,(0,t)\,} \right\}\,} \right]\,\,, \\ \end{aligned}$$

96 $$\begin{aligned} \hbar ^{2} :\,\eta _{2} \,(0,t) &= \frac{{\vartheta _{2} }}{\varpi }\,\sin \,\varpi \,t + L_{T}^{{ - 1}} \,\left[ {\frac{1}{{S^{2} + \varpi ^{2} \,}}} \right.L_{T} \left\{ {\Re _{1} \,\eta _{1} \,(0,t)} \right. + \Re _{2} \,\eta _{0} \,(0,t)\, + \,\,i\,f_{0} \,\eta _{1} (0,t)\, + \,\,(g_{1} + i\,f_{1} )\,\,\eta _{{1t1}} \,(0,t) \\&\quad + 2\,(g_{2} + i\,f_{2} )\,\,\eta _{1} (0,t)\,\eta _{0} (0,t) + \,\,\,3(g_{3} + i\,f_{3} )\,\eta _{0}^{2} (0,t)\,\eta _{1} (0,t) + \,(g_{4} + i\,f_{4} )\,\left( {\eta _{1} (0,t)\,\,\eta _{{0t}} \,(0,t) + \eta _{0} (0,t)\,\,\eta _{{1t}} \,(0,t)} \right) \\&\quad + (g_{5} + i\,f_{5} )\,\left( {\,\eta _{0}^{2} (0,t)\,\eta _{{1t}} (0,t) + 2\eta _{0} (0,t)\,\eta _{1} (0,t)\eta _{{0t}} (0,t)} \right) + \,\,\,g_{6} \,\left( {\eta _{1} (0,t)\,\,\eta _{{0tt}} (0,t) + \eta _{0} (0,t)\,\,\eta _{{1tt}} \,(0,t)} \right) \\&\quad \left. {\left. { + g_{7} \left( {\,\eta ^{2} _{0} (0,t)\,\,\eta _{{1tt}} \,(0,t) + 2\,\eta _{0} (0,t)\,\eta _{1} (0,t)\eta _{{0tt}} (0,t)} \right)\,} \right\}\,} \right]\,\, \\ \end{aligned}$$

We will substitute from Eq. (94) into Eq. (95) and remove the secular terms. For this, it is better to ignore the coefficients of the functions $\cos \,\,\varpi \,\,t$ and $\sin \,\,\varpi \,\,t$ at all phases; solving the two equations simultaneously produces the values of ${\mathfrak{R}}_{1}$ and ${\vartheta }_{0}$.

97 $$\Re_{1} = - \frac{{3f_{1} }}{{g_{5} }}(g_{3} - \varpi^{2} g_{7} )\,,\,\,\,\,\,\,\,\vartheta_{0}^{2} = - \,\frac{{4\varpi^{2} \,f_{1} }}{{g_{5} }},\,\,\,\,\,$$

The periodical solution at this point in time is provided by

98 $$\eta _{1} = \mathchar'26\mkern-10mu\lambda _{0} + \mathchar'26\mkern-10mu\lambda _{1} \sin \,\varpi t + \mathchar'26\mkern-10mu\lambda _{2} \cos \,\varpi t + \mathchar'26\mkern-10mu\lambda _{3} \sin 2\varpi t + \mathchar'26\mkern-10mu\lambda _{4} \cos 2\varpi t + \mathchar'26\mkern-10mu\lambda _{5} \sin \,3\varpi t + \mathchar'26\mkern-10mu\lambda _{6} \cos \,3\varpi t,$$

where $\mathchar'26\mkern-10mu\lambda _{i} ,i = 0,1,2,...,6$ are quantities identified from the framework.

Following the abovementioned procedures, one gets:

99 $$\vartheta_{1} = \frac{{\vartheta_{0}^{2} }}{{96\varpi^{6} f_{1} + 72\varpi^{4} g_{5} \vartheta_{0}^{2} }}\left( \begin{gathered} 32\varpi^{4} f_{1} g_{4} + (9\varpi^{2} f_{3} g_{1} - 9\varpi^{2} f_{1} g_{3} + 24\varpi^{2} g_{2} g_{4} + 9\varpi^{4} f_{1} g_{7} )\vartheta_{0} + (48f_{2} f_{3} - 48g_{2} g_{3} \hfill \\ + 24\varpi^{2} g_{4} g_{5} + 48\varpi^{2} g_{3} g_{6} + 48\varpi^{2} g_{2} g_{7} - 48\varpi^{4} g_{6} g_{7} )\vartheta_{0}^{2} + (12f_{3} f_{5} - 12g_{3} g_{5} + 18\varpi^{2} g_{5} g_{7} )\vartheta_{0}^{3} \hfill \\ + \varpi^{2} f_{0} (64f_{2} + 3f_{5} \vartheta_{0} ) - 8\varpi^{2} f_{4} (4\varpi^{2} g_{1} + 3(\vartheta_{0} f_{2} + f_{5} \vartheta_{0}^{2} )) + (64\varpi^{2} g_{2} - 64\varpi^{4} g_{6} + 3\varpi^{2} g_{5} \vartheta_{0} )\Re_{1} \hfill \\ \end{gathered} \right)$$

Therefore, the following is how the bounded approximation of the equation of motion is provided in Eq. (86)

100 $$\eta = \lim_{h \to 1} (\eta_{0} + \hbar \eta_{1} + ...)$$

In reality, the restricted estimated solution in Eq. (100) requires that the trigonometric function arguments be real numbers. It can be concluded that the extended frequency fulfills a particular characteristic equation when Eqs. (89) and (97) are combined. The analysis revealed that, at the extended frequencies, this equation resembles a polynomial of the second degree. This equation can be solved to determine the value.

101 $$\varpi = \sqrt {\frac{{3f_{1} g_{3} - \varpi^{2} g_{5} }}{{3f_{1} g_{7} - g_{5} }}}$$

The following figure is a graph of a structure receiving the following specifics:

$$\begin{gathered} \lambda_{1} = 1,\,\lambda_{2} = 0.5\,,\,\,\mu_{1} = 3,\,\,\mu_{2} = 1,\,\,\rho_{1} = 0.2,\,\,\rho_{2} = 4,\,\,V_{1} = 0.3,\,\,V_{2} = 0.5,\,\,\alpha_{1} = 0.2,\,\, \hfill \\ \alpha_{2} = 0.5,\,\,\alpha_{3} = 15,\,\,a = 1,\,\,b = 7,\,\,R = 4,\,\,\kappa = 0.1,\,\,\varepsilon_{1} = 7,\,\,\varepsilon_{2} = 0.2\,\,{\text{and }}\,{\text{H}}_{{0}} = 4. \hfill \\ \end{gathered}$$

**Figure 14:**
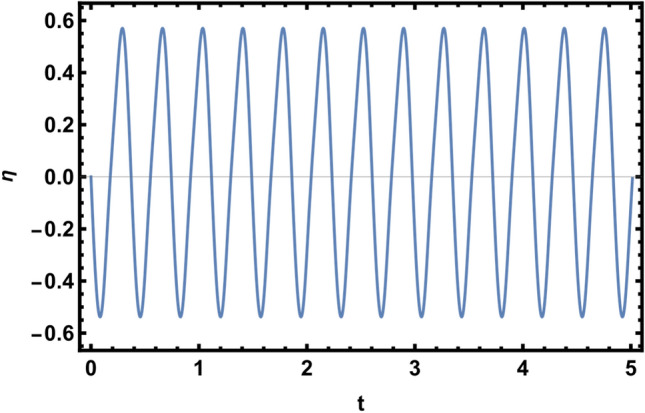
Disturbed response as specified in Eq. (100).

The computations revealed that the value of the expanded frequencies exists and equals $\varpi = 2.69$. The disturbing response from Eq. (100) will be plotted in Fig. 14. This figure demonstrates the periodic behavior of the profile of the surface displacement. The prior behavior results from the secular terms of the non-homogeneous parts of the controlling equation of motion of the surface waves that is cancelled out.

## Concluding remarks

In this study, we have examined the linear and nonlinear stability of a vertical cylindrical interface that separates between two homogeneous incompressible dielectric viscoelastic liquids. The inner and outer liquids are occupied by a non-Newtonian Oldroyd-B type. The Oldroyd-B fluid possess both relaxation, retardation times in addition a viscosity parameter. The system is acted upon by an unchanged longitudinal electric field. A basic construction of VPF is utilized to reduce the mathematical workload. Numerous specific cases are represented based the selected data. Through porous media, Darcy's law controls the flow. The permeability of the two fluids is referred to simply as for convenience. Darcy's law, an experimental hypothesis that describes the creeping flow of Newtonian liquids through porous media, is supported by scientific evidence. Additionally, MHT is reflected with the shortened formulation of Hsieh^38^ and^39^. The nonlinear approach contributes primarily to the solution of the fundamental linear equations of motion and using the relevant suitable nonlinear border circumstances. This process produces a nonlinear characteristic equation of the surface displacement of the surface waves. The dimensionless procedure exposes a collection of dimensionless physical numbers, in addition to our prior work, see Moatimid and Mostapha^60^. A linear dispersion relation is obtained by disregarding the nonlinear elements. The linear stability standards are judged via the Routh-Hurwitz theory. To confirm the theoretical outcomes, numerical estimations are carried out. A series of diagrams are depicted in order to confirm similar findings to those reported in the literatures. For the nonlinear characteristic equation, a novel approach is utilized to achieve the stability benchmarks. Consequently, the stability standards are theoretically and numerically validated. Our upcoming study will address these forms non-Newtonian models in consideration due to the practical applications of the various structures of non-Newtonian prototyping as progress works. Furthermore, particular linear stability non-Newtonian standards were explored along with our earlier articles^10, 46, 50–52^. Therefore, away from the simplified formulation of Hsieh’s formula^38^ and[39[, the future works will adopt the energy as well as the concentration equations. Without any doubt, this approach reveals several parameters concerning the MHT phenomenon. The nonlinear stability approaches will be emphasized in order to get a higher degree of precision. Overall, the stability zones are enhanced in the nonlinear instability approach in distinction with those in the linear approach. For more convenience, the stabilizing/destabilizing influences of various physical parameters in the problem at hand, may be summarized throughout the following:

- **In light of the linear approach**

**Table:** 

Physical parameters	Behavior
Laplace numeral $L_{a}$	U
Reynolds numeral $R_{e}$	U
Weber numeral $W_{e}$	U
Darcy numeral $D_{a}$	S
Elasticity numeral $E_{l}$	U
MHT constant $\alpha_{1}$	U

- **In light of the nonlinear approach**

**Table:** 

Physical parameters	Behavior
Viscosities of liquid $\mu_{l}$	U
MHT constant $\alpha_{1}$	S
MHT constant $\alpha_{3}$	S
Permeability porosity $\kappa$	S
Radius $R$	Dual role

- Additionally, throughout the nonlinear stability methodology, the graph of the surface displacement has been established in a uniform formula after the elimination of the secular terms.

As we make progress, the following topics will be addressed in later papers:

- In addition to providing the existence of the energy and concentration equations, Hsieh's simple approach will no longer be used.
- Various nanofluids with the numerous non-Newtonian fluids will be implemented.
- Different stability issues in a plane geometry will be addressed in light of the importance of numerous actual engineering implementations.
- A time-varying exterior fields will be supplied.
- An innovative method will be used to examine the stability methodology in light of the non-perturbative approaches in evaluating the nonlinear partial differential equations..

## Supplementary Information

Supplementary Information.

## Data Availability

All data generated or analyzed during this study are included in this manuscript.

## English symbols

$$(r,\,\theta ,\,z)$$: Cylindrical coordinates
$a\,\,$: Inner cylindrical radius $l$
$b\,\,\,$: Outer cylindrical radius $l$
$R\,$: Cylindrical undisturbed radius $l$
$T_{a}$: Temperature of the inner rigid cylinder $K$
$T$: Temperature of the interface $K$
$T_{b}$: Temperature of the outer rigid cylinder $K$
$$L_{{\rho_{1} }}$$: Latent heat $$ml^{2} t^{ - 2}$$
$K_{l} \,$: Heat conductivity of the inner flow $ml^{5} t^{ - 3} K^{ - 1}$
$K_{g} \,$: Heat conductivity of the outer flow $ml^{5} t^{ - 3} K^{ - 1}$
$G\,$: Equilibrium state of heat fluxes $ml^{4} t^{ - 3}$
$$g$$: Gravitational acceleration $$lt^{ - 2}$$
$\underline{\Upsilon } \,$: Darcy resistance for an Oldroyd-B $ml^{ - 2} t^{ - 2}$
$\underline{e}_{r}$: Unit vector along with $r -$ path
$\underline{e}_{\theta }$: Unit vector along with $\theta -$ path
$\underline{v}_{l} \,$: Liquid fluid velocity $lt^{ - 1}$
$\underline{v}_{g}$: Gas fluid velocity $lt^{ - 1}$
$c.\,c.$: Complex conjugate of the preceding terms

## Greek symbols

$\rho_{l} \,$: Inner liquid density $ml^{ - 3}$
$\rho_{g} \,$: Outer gas density $ml^{ - 3}$
$\nu_{l}$: Inner Darcy’s coefficient $ml^{ - 3} t^{ - 1}$
$\nu_{g}$: Outer Darcy’s coefficient $ml^{ - 3} t^{ - 1}$
$\varepsilon_{l}$: Inner liquid permittivity
$\varepsilon_{g}$: Outer gas permittivity
$\sigma \,\,$: Surface tension coefficient $mt^{ - 2}$
$\mu \,$: Fluid viscoelasticity coefficient $ml^{ - 1} t^{ - 1}$
$\omega$: Frequency of surface waves $t^{ - 1}$
$\omega_{r} \,$: Real portion of the frequency of surface waves $t^{ - 1}$
$\omega_{i} \,$: Imaginary portion of the frequency of surface waves $t^{ - 1}$
$$\eta \,\,\,$$: Interfacial deflection $$l$$
$\delta$: Primary amount of interfacial deflection
$\kappa$: Permeability of media $l$
$\lambda_{1} ,\,\lambda_{2} \,$: Relaxation and retardation periods, of the Oldroyd-B fluid, respectively $t$
$\mu_{l} \,,\,\,\mu_{g}$: Viscosities of liquid and gas, respectively $ml^{ - 1} t^{ - 1}$
$$\underline{\underline{\tau }} \,\,$$: Extra tensor $$ml^{ - 1} t^{ - 2}$$
$$\xi \,$$: Permeability $$l^{2}$$
