# Supplementary material for: Nonlinear EHD instability of two viscoelastic fluids under the influence of mass and heat transfer
Source: Sci Rep. 2023 Jan 7;13:357. doi: 10.1038/s41598-023-27410-z (PMC9825410; doi:10.1038/s41598-023-27410-z)
Supplement: Supplementary file 1 — Supplementary Information. [file 41598_2023_27410_MOESM1_ESM.docx]

**Appendix**

The following is a list of the quantities used in Eq. (47):

$$\begin{matrix} a_{1}=\frac{1}{E \zeta_{1}}\left(\begin{matrix} \frac{-\alpha_{1}}{k}(I_{1}(ak)K_{0}(kR)+I_{0}(kR)K_{1}(ak))-\frac{\alpha_{1}}{k}(I_{1}(bk)K_{0}(kR)+I_{0}(kR)K_{1}(bk))- \\ k\mu_{1}(I_{1}(ak)K_{0}(kR)+I_{0}(kR)K_{1}(ak)) \\ -k\mu_{1}(I_{2}(kR)K_{1}(ak)+I_{1}(ak)K_{2}(kR))-\frac{\nu_{1}}{k}(I_{1}(ak)K_{0}(kR)+I_{0}(kR)K_{1}(ak))+ \\ \frac{2k\alpha_{1}\lambda_{1}\mu_{1}}{\rho_{1}}(I_{1}(ak)K_{0}(kR)+I_{0}(kR)K_{1}(ak) \\ +\frac{2\alpha_{1}\lambda_{1}\mu_{1}}{R\rho_{1}}(-I_{1}(kR)K_{1}(ak)+I_{1}(ak)K_{1}(kR))-\frac{2k\alpha_{1}\lambda_{2}\mu_{1}}{\rho_{1}}(I_{1}(ak)K_{0}(kR)+I_{0}(kR)K_{1}(ak))+ \\ \frac{2\alpha_{1}\lambda_{2}\mu_{1}}{R\rho_{1}}(I_{1}(kR)K_{1}(ak)+I_{1}(ak)K_{1}(kR)) \end{matrix}\right)+ \\ \frac{\nu_{2}}{E \zeta_{2}k}(I_{1}(bk)K_{0}(kR)+I_{0}(kR)K_{1}(bk)) \end{matrix}$$

$b_{1}=-\frac{V_{1}\rho_{1}}{E \zeta_{1}}(I_{1}(ak)K_{0}(kR)+I_{0}(kR)K_{1}(ak))+\frac{V_{2}\rho_{2}}{E \zeta_{2}}\left(I_{1}(bk)K_{0}(kR)+I_{0}(kR)K_{1}(bk)\right),$ $\begin{matrix} a_{2}=\frac{1}{E \zeta_{1}}\left(\begin{matrix} -kV_{1}\mu_{1}\left(I_{1}(ak)K_{0}(kR)+I_{0}(kR)K_{1}(ak)+I_{2}(kR)K_{1}(ak)+I_{1}(ak)K_{2}(kR)\right)- \\ \frac{V_{1}\nu_{1}}{k}\left(I_{1}(ak)K_{0}(kR)+I_{0}(kR)K_{1}(ak)\right) \end{matrix}\right)+ \\ \frac{V_{2}\nu_{2}}{E \zeta_{2}k}(I_{1}(bk)K_{0}(kR)+I_{0}(kR)K_{1}(bk)), \end{matrix}$

$$b_{2}=b_{21}E_{0}^{2}+b{}_{22},$$

$$b_{21}=\frac{1}{E}\left[\begin{matrix} (\varepsilon_{1}-\varepsilon_{2}) \\ \left(\begin{matrix} (I_{1}(ak)K_{0}(kR)+I_{0}(kR)K_{1}(ak))(I_{1}(bk)K_{0}(kR)+I_{0}(kR)K_{1}(bk))\varepsilon_{1}-(I_{1}(ak)K_{0}(kR)+ \\ I_{0}(kR)K_{1}(ak))(I_{1}(bk)K_{0}(kR)+I_{0}(kR)K_{1}(bk))\varepsilon_{2} \end{matrix}\right) \\ /\left((I_{1}(bk)K_{0}(kR)+I_{0}(kR)K_{1}(bk)) \zeta_{1}\varepsilon_{1}-(I_{1}(ak)K_{0}(kR)+I_{0}(kR)K_{1}(ak)) \zeta_{2}\varepsilon_{2}\right) \end{matrix}\right],$$

$$b_{22}=-\frac{V_{1}v_{1}\rho_{1}}{E \zeta_{1}}\left(I_{1}(ak)K_{0}(kR)+I_{0}(kR)K_{1}(ak)\right)+\frac{V_{2}v_{2}\rho_{2}}{E \zeta_{2}}(I_{1}(bk)K_{0}(kR)+I_{0}(kR)K_{1}(bk))$$

$\begin{matrix} a_{3}=\frac{T}{E R^{2}}-\frac{1}{E \zeta_{1}}\left(\begin{matrix} \frac{-k\alpha_{1}\mu_{1}}{\rho_{1}}\left(I_{1}(ak)K_{0}(kR)+I_{0}(kR)K_{1}(ak)+I_{2}(kR)K_{1}(ak)+I_{1}(ak)K_{2}(kR)\right)- \\ \frac{\alpha_{1}\nu_{1}}{k\rho_{1}}\left(I_{1}(ak)K_{0}(kR)+I_{0}(kR)K_{1}(ak)\right) \end{matrix}\right)+ \\ \frac{\alpha_{1}\nu_{2}}{E \zeta_{2}k\rho_{2}}(I_{1}(bk)K_{0}(kR)+I_{0}(kR)K_{1}(bk)), \end{matrix}$ $b_{3}=\frac{V_{1}\alpha_{1}}{E \zeta_{1}}(I_{1}(ak)K_{0}(kR)+I_{0}(kR)K_{1}(ak))+\frac{V_{2}\alpha_{2}}{E \zeta_{2}}(I_{1}(bk)K_{0}(kR)+I_{0}(kR)K_{1}(bk)),$

$$\begin{matrix} a_{4}=\frac{1}{E \zeta_{1}}\left(\begin{matrix} 2kV_{1}\lambda_{1}\mu_{1}(I_{1}(ak)K_{0}(kR)+I_{0}(kR)K_{1}(ak))-\frac{2V_{1}\lambda_{1}\mu_{1}}{R}(I_{1}(kR)K_{1}(ak)-I_{1}(ak)K_{1}(kR))- \\ 2kV_{1}\lambda_{2}\mu_{1}(I_{1}(ak)K_{0}(kR)+I_{0}(kR)K_{1}(ak)) \\ -\frac{2V_{1}\lambda_{2}\mu_{1}}{R}(I_{1}(kR)K_{1}(ak)-I_{1}(ak)K_{1}(kR))-\frac{V_{1}\rho_{1}}{k}(I_{1}(ak)K_{1}(kR)+I_{0}(kR)K_{1}(ak)) \end{matrix}\right)+ \\ \frac{V_{2}\rho_{2}}{E \zeta_{2}k}(I_{1}(bk)K_{0}(kR)+I_{0}(kR)K_{1}(bk)), \end{matrix}$$

$$a_{5}=\frac{T}{E R},$$

$$\begin{matrix} E=\frac{1}{\zeta_{1}}\left(\begin{matrix} 2k\lambda_{1}\mu_{1}\left(I_{1}(ak)K_{0}(kR)+I_{0}(kR)K_{1}(ak)\right)-\frac{2\lambda_{1}\mu_{1}}{R}\left(I_{1}(kR)K_{1}(ak)-I_{1}(ak)K_{1}(kR)\right)- \\ 2k\lambda_{2}\mu_{1}\left(I_{1}(ak)K_{0}(kR)+I_{0}(kR)K_{1}(ak)\right) \\ +\frac{2\lambda_{2}\mu_{1}}{r}\left(I_{1}(kR)K_{1}(ak)-I_{1}(ak)K_{1}(kR)\right)-\frac{\rho_{1}}{k}\left(I_{1}(ak)K_{0}(kR)+I_{0}(kR)K_{1}(ak)\right) \end{matrix}\right)+ \\ \frac{\rho_{2}}{\zeta_{2}k}(I_{1}(bk)K_{0}(kR)+I_{0}(kR)K_{1}(bk)), \end{matrix}$$

**$\zeta_{1}=I_{1}(kR)K_{1}(ak)-I_{1}(ak)K_{1}(kR)$** and **$\zeta_{2}=I_{1}(kR)K_{1}(bk)-I_{1}(bk)K_{1}(kR)$.**
